# Raman Spectroscopy for Nitrate Detection in Water: A Review of the Current State of Art

**DOI:** 10.1021/acsmeasuresciau.5c00016

**Published:** 2025-07-28

**Authors:** Lorenzo Luciani, Antonio Nocera, Michela Raimondi, Gianluca Ciattaglia, Susanna Spinsante, Ennio Gambi, Rossana Galassi

**Affiliations:** † Scuola di Scienze e Tecnologie, Divisione di Chimica, Università di Camerino, Camerino 62032, Italy; ‡ Dipartimento di Ingegneria dell’Informazione, 9294 Università Politecnica delle Marche, Ancona 60131, Italy

**Keywords:** Raman spectroscopy, nitrate detection, SERS, FERS, machine learning, water analysis, applied spectroscopy, Raman data processing

## Abstract

The contamination of natural basins by agricultural or industrial activities, and the growing need for potable water due to climate changes accelerate the drive to find versatile, fast, practical, and easy-to-use methods for water analysis. A potentially versatile technique suitable for water analysis is Raman Spectroscopy (RS). Featured by good resolution but low sensitivity, RS detects molecular vibrational modes of an analyte in water. Nitrate is an indicator of chemical and/or biological pollution, it displays Raman active vibrational modes affected by the interaction with other systems in solution, allowing a wide range of applications. Concerning Nitrate analysis in water, a general introduction to the Raman effect and the basic instrumentation were herein discussed. RS is a potential solution to wastewater analysis. This review first reports the theoretical background of the technique and its basic working principles, then, the state-of-the-art scientific contributions related to Nitrate detection are investigated with a particular interest in the instrumental setup and the chemometric techniques employed to improve its sensitivity. In the studies hereby considered, instrumental setup (for example, laser frequency, laser power, acquisition times) and different technical solutions (for example, micro- versus macro-Raman instruments) to increase the technique’s sensitivity on Nitrate detection are described. Concisely, the use of deep-UV lasers, optically active Surface-Enhanced Raman Spectroscopy (SERS) or Fiber-Enhanced Raman spectroscopy (FERS) equipment, coupled with instrumental settings, i.e. acquisition time, variable temperature of acquisition, use of special sampling apparatus (cuvettes or immersion probes), or with ion exchange resins for analyte enrichment, have been reported. Remarkably, examples of large data correction of unwanted fluorescence by mathematical processing or chemical quenching were reported too, suggesting solutions for the Raman analysis of wastewaters. Finally, a short digression on Machine Learning (ML) applied to RS is proposed, showing the promising results reported in other fields. Data-driven methods could be a solution to improve the low sensitivity of the RS for Nitrate detection. Hence, an approach of ML methods for the typical RS spectra processing (spike removal, baseline correction, fluorescence curve elimination, instrumental noise correction) was hereby mentioned, suggesting an improvement in the detection capability of Nitrate ion in water.

## Graphical abstract

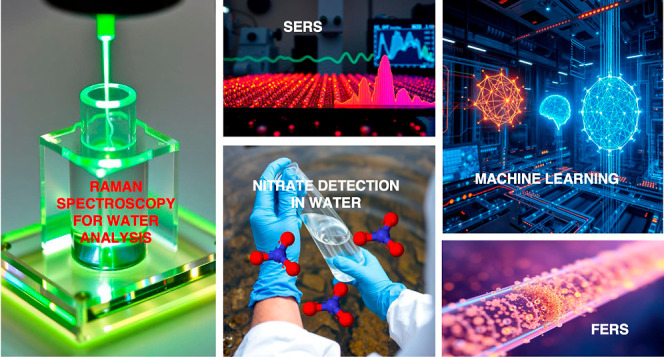

## Introduction

The quality of natural water resources can be influenced by natural effects or anthropological activities, and the most pervasive issue is inadequate access to clean water and sanitation.^1^ The first step in facing this problem is determining the overall composition and detecting specific pollutants. Various instrumental methodologies are available, but mostly they are lab-centered.^2^ Particularly, traditional methods applied for water quality detection^3^ involve enrichment analysis,^4^ volumetric analysis,^5^ electrochemical analysis,^6^ spectrophotometry,^7^ atomic emission spectrum analysis, fluorimetric spectroscopy,^8^ Atomic Absorption Spectroscopy (AAS),^9^ infrared spectroscopy,^10^ and chromatography.^11^ These classic instrumental methods can be applied both to detect pollutants and for quantitative measurements in water; however, most of these methods feature elaborate sample preparations and no direct measurements. The propulsive need to get fast measurements without complicated sample handling and preparation inspired the research of new fast, reliable methods of measurement. The development of a wide range of optical technologies and signal processing techniques sheds light on RS as a modern method for the detection of substances in water. Recent technological innovation and new detection devices and optical fibers gave a strong impulse to water analysis by RS.^12–14^ Cn.^15^ Moreover, it offers highly structured information, high resolution with narrow bandwidths, and minimal sample preparation. It is often coupled to fiber-based optics to achieve in situ analysis by remote control, affording applications for continued monitoring of flow processes.^16^ Considering all these properties, upon the dawn of environmental awareness and regulation, RS was soon evaluated for contaminant water analysis. In this review, among all the possible contaminants, nitrate has been selected as a case study for the relevance of its identification in terms of water analysis and pollution detection.^17^ Some fundamental concepts of RS are presented, along with current technological approaches for analyzing the Raman-active nitrate anion.^18^ Considering the recent widespread adoption of data optimization through artificial intelligence,^19^ a brief discussion is also included on cases where ML data processing has facilitated Nitrate detection by RS.

### Basic Concepts on Raman Spectroscopy

The Raman effect consists of components of scattered light obtained from a Raman-active transparent sample caused by the irradiation with monochromatic Ultra Violet (UV), visible, or Near Infrared Radiation (NIR) light from a laser at frequency ν_0_; the scattered light is mainly due to the input frequency ν_0_ (Rayleigh scattering) and also, to a much lesser extent, in narrow bands at other discrete frequencies, namely the Raman shifts. These latter are at both lower and higher frequencies than that of the laser, with the lower frequency bands termed Raman Stokes bands (*h*ν_0_ – *h*ν), and the higher frequency bands (*h*ν_0_ + *h*ν) are termed Raman anti-Stokes bands (Figure 1). Removing the Rayleigh component, the Raman Stokes bands are due to the vibrational modes of Raman active species transitioning from the vibrational ground state to the first excited vibrational state.

**Figure 1:**
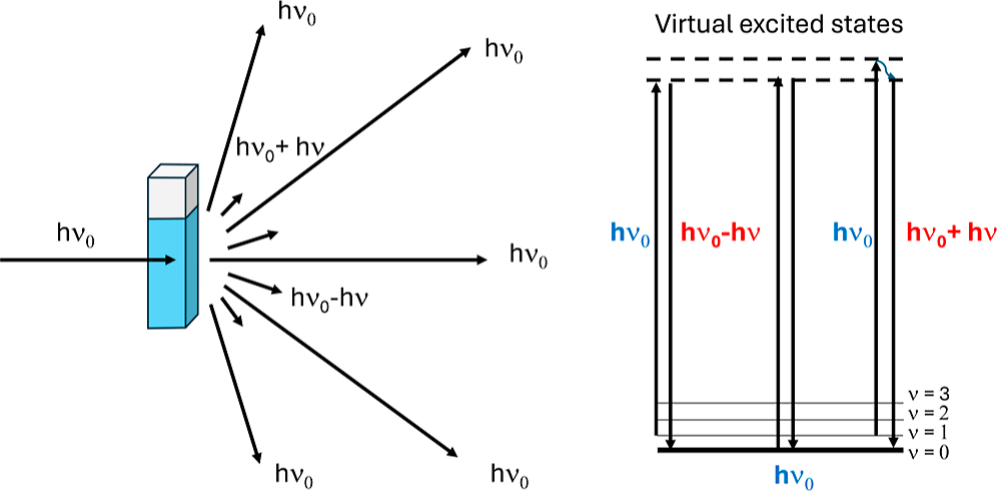
(Left) Raman scattering by a water solution upon laser excitation (*h*ν_0_); (right) energy diagram for elastic Rayleigh (*h*ν_0_) and inelastic Stokes (*h*ν_0_ – *h*ν) and anti-Stokes (*h*ν_0_ + *h*ν) scatterings.

However, considering the thermal Boltzmann distribution, the ground vibrational energy level (υ_0_) is largely more populated than a higher vibrational energy level (e.g., υ_1_) near room temperature, making the anti-Stokes dramatically less intense than the Stokes shifts. A Rayleigh scattered photon is emitted upon 10^4^ excitation photons, while one Stokes Raman scattered photon occurs for each 10^10^ excitation photon. A fundamental difference between vibrational energy absorption (Infrared Spectroscopy) and Raman scattering can be discussed in a typical solution for an absorption experiment where the concentration *C* is 10^–3^ M, ϵ = 1000 M^–1^ cm^–1^ absorbs 90% of the incident light over a path length of 1 cm, nonetheless, only about 1 over 10^10^ incident photons undergoes Raman scattering. Therefore, the Raman effect is relatively faint, requiring the scattered light to be captured by a highly sensitive Charge-Coupled Device (CCD) detector and analyzed through a spectrograph configured to detect and record only radiation at frequencies lower than that of the excitation laser (Stokes shifts). Dispersive RS is a fundamental vibrational technique commonly used for solids or solutions, which are typically prepared by dissolving the pure substance in water at high concentrations. Hence, the spectrum contains vibrational bands due to the analyte and bands from the Raman-active vibrational modes of water. By subtracting the spectrum of pure water from the spectrum of the solution, the resulting spectrum, in the simplest case, approximately matches that of the solute, and the intensity of the bands will be linearly related to the concentration of the analyte in the aqueous solution. Remarkably, the most controversial discussion in RS applications is the instrumental setup. Instrumental factors strongly affect the Raman intensity. The intensity of the Raman bands (*I*) depends on many factors,^20,21^ the signal strength is directly proportional to the fourth power of the laser frequency (*f*), hence inversely proportional to the fourth power of the laser wavelength (λ), is directly proportional to the intensity of the laser radiation (*I*_L_), to the density of scattering molecules (*N*), and related to the sample nature by the polarizability change (δα) upon the interaction with an electric field (δ*q*), as described in eq 1

1 $$I∼f^{4}\times I_{L}\times N\times \left(\frac{\delta \alpha }{\delta q}\right)∼\left(\frac{1}{\lambda^{4}}\right)$$

The intensity of the scattered photons depends on the acquisition time too, adding another purely instrumental parameter to the quoted variables.^21^ Hence, the first two terms of eq 1 are the most significant for the intensity of the bands addressing a relationship between the intensity and the adopted laser. Moreover, the frequency of scattered light depends on the frequency of the laser; hence, short-wavelength excitations promote high-energy scattered frequencies. Nevertheless, the choice of high-frequency lasers may complicate the analysis, causing the molecules to transit from a “virtual” excited state to an “actual” excited state, leading to unwanted laser-induced fluorescence, indeed, as much as the laser’s energy, as likely this event is. Hence, high-frequency (low-wavelength) lasers increase the scattered light intensity and the probability of observing unwanted fluorescence or even sample detriment. Hence, the choice of the input laser mostly affects the sensitivity of the Raman instrument, even though many technological solutions can be selected in the instrumental setup.

### Instrumental Components

A basic Raman spectrophotometer comprises five components: an excitation source, a light collection system, a monochromator, a detector, and a data processing system (Figure 2).

**Figure 2:**
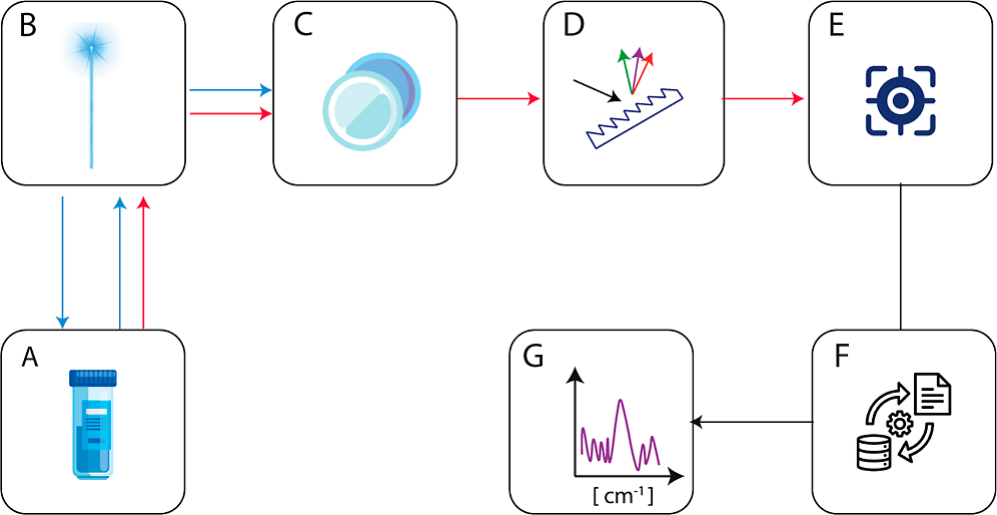
Schematic blocks diagram for a generic Raman Spectrophotometer. A = vial with the water solution, B = laser source, mirror, C = optical filter, D = spectrograph, E = detector, F = data processor, G = spectrum.

A Raman excitation source should feature a narrow monochromatic bandwidth and high power^22,23^ making lasers as suitable and convenient sources.^24^ The most common laser output characteristics are the wavelength, the emission bandwidth and monochromaticity, the spatial collimation, the output power, the coherence and the polarization. The wavelength range extends from the UV to the NIR and does not account for other more exotic systems that provide access from the soft-X-ray spectral region (less than 10 nm) to the Far-Infrared Radiation (FIR) (more than 100 μm). The lasing wavelength (herein called ν_0_) is determined by the laser gain medium (for example He: Ne (Helium–Neon); He–Cd (Helium–Cadmium); Ar (Argon); Nd: YAG (Neodinium-YAG, with YAG is a crystal of Y_3_Al_5_O_12_)), which provides the optical transition. The wide range of wavelengths is attributable to the large variety of available gain media. Furthermore, nearly all laser wavelengths can be converted or shifted to an alternative wavelength from UV to NIR spectral regions. The monochromator is used to discriminate scattered light based on wavelength and is the most characterizing component of a Raman spectrophotometer. Gratings and multistage monochromators are often chosen for dispersive RS, while the Michelson interferometer is considered for multiplexing not-dispersive Fourier Transformer (FT) Raman spectrophotometer. Multiplexing is a technique of “composition” and “decomposition” of light components that allows sending multiple digital data flows in a single signal using a single communication channel. However, the overwhelming shot noise of the Rayleigh line over the weak Raman lines requires removal of elastic light scattering. Effectively, the noise redistribution characteristic of Fourier spectroscopy spreads the overwhelming shot noise of the “Rayleigh line” over the weak Raman lines and makes them unobservable^25^ since the equivalent stray light rejection of a shot-noise-limited Michelson interferometer is of the order of 10^–3^ instead of the needed and desirable 10^–10^. The detector is the device for collecting signals from the spectrograph and inputting data into the data processor. CCDs are mostly applied in Raman instruments because of the extremely low Raman cross sections of nonresonant molecules, hence an efficient signal-enhancement technique, or long integrations using multichannel detection to have monolayer sensitivity, is needed.^26^ A CCD detector consists of a metal oxide semiconductor capacitor array on a thin silicon substrate.^27^ CCDs exhibit high sensitivity, low detection noise, multichannel array, and high quantum efficiency. For better performance, a CCD needs to be cooled and a liquid-nitrogen-cooled CCD detector affords high-quality multichannel even in unenhanced Raman scattering spectra. Afterward, data compression and noise removal actions should be included to improve the feasibility of information from the data. Data processing involves the adjustment of the baseline and the removal of eventual fluorescence; for example, the correction of a set of data by subtracting a polynomial baseline to model the fluorescence affording to the “clean” Raman peaks is reported in Figure 3.

**Figure 3:**
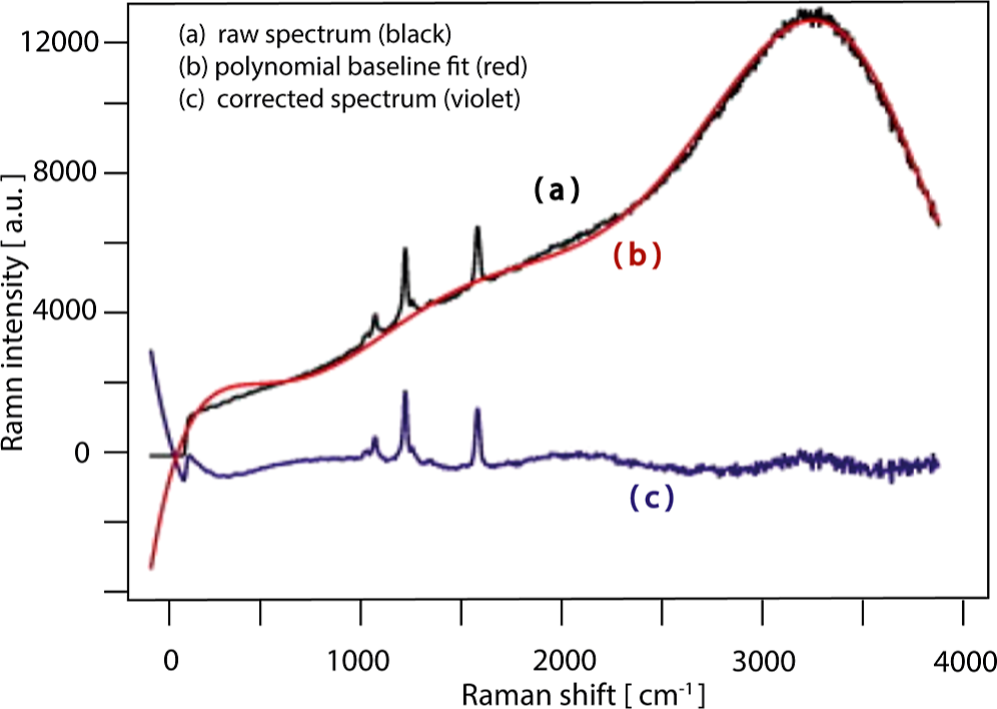
Removal of the fluorescence background in confocal Raman microspectroscopy. (a) The raw Raman spectrum (black) shows fluorescence interference. (b) Spectrum correction by a polynomial baseline to model the fluorescence (red). (c) The corrected spectrum (violet) reveals the “clean” Raman signals. Reproduced or adapted with permission from ref 28. Copyright 2012, Elsevier.

This introductory discussion emphasizes that RS involves weak signals and that the instrumental configuration is essential for obtaining a diagnostic spectrum. The low sensitivity can sometimes be addressed through the use of powered lasers and extended acquisition times, low-temperature CCDs, as well as by incorporating additional instrumental components such as in SERS, which enhances Raman signals through molecule absorption on rough metal or other material surfaces, or by utilizing specialized optical fibers in the setup, as seen in FERS.

### Raman Detection of the Nitrate Anion

Some anions like nitrate, nitrite, sulfates, chromate, cyanide, fluorides, and phosphates are significant environmental concerns due to their potential for bioaccumulation and harmful effects on living organisms.^29^ Furthermore, anions are regarded as water pollutants that cause harmful effects such as eutrophication, which can ultimately result in the death of aquatic organisms.^30^ Excessive exposure to nitrate and nitrite poses risks for human health, because it causes the formation of highly carcinogenic species like nitrosamines.^31^ Focusing on nitrate detection in water, it is essential in various contexts, ranging from natural and potable waters to industrial, urban, and agricultural water source.^32^ Currently, various techniques are employed for nitrate detection, including potentiometric, chromatographic, atomic absorption, and colorimetric methods.^33^ Due to its inherent ability to simultaneously detect multiple species in solution with sufficient resolution, RS can be a suitable technique for nitrate detection.^34^ Nitrate is a polyoxoanion, and its salts are highly soluble in water. Additionally, it exhibits notable anisotropic molecular polarizability, which enables Raman inelastic scattering through interaction with UV or visible laser light.^35^ RS can serve as a valuable method for water analysis due to its inherent ability to detect multiple species simultaneously with sufficient resolution. Free nitrate adopts a trigonal planar geometry, exhibiting *D*_3*h*_ symmetry. It comprises three equivalent N–O bonds, each with partial double-bond character, and carries an excess negative charge distributed over the oxygen atoms. The symmetry operations corresponding to the *D*_3*h*_ point group are listed in the character table^36^ describing the Raman active vibrational modes.^35^

The Raman spectrum of nitrate is dominated by the double degenerate fundamental in-phase symmetric N–O stretching near 1043 cm^–1^, (ν_1_), while the out-of-plane deformation (ν_2_) near 830 cm^–1^, the out-of-phase N–O stretch (ν_3_) near 1370 cm^–1^ and the in-plane bend (ν_4_) near 723 cm^–1^ are the other vibrational modes. In an ideal trigonal planar structure like the free nitrate ion, the only Raman-active vibration permitted is the ν_1_, while the ν_3_ and the ν_4_ are both Raman and IR active. Nevertheless, the solvation environment and/or the formation of cation–anion couples affect the symmetry and partially localize the negative charge,^37^ changing the symmetry from *D*_3*h*_ of the free anion to at least *C*_2ν_, deactivating ν_1_ and activating ν_2_.^38^ The nitrate anion may remain planar, but the N–O bonds involved in interactions are inequivalent compared to the other two N–O bonds. Contextually, in a structure with a symmetry better described as *C*_2ν_, the ν_1_ and ν_2_ vibrational modes become both IR and Raman active, and the double degeneracy is lost. Ab initio calculation displayed that ν_3_ and ν_4_ are split by about 50 and few cm^–1^, respectively.^39^ Therefore, examining the Raman peak intensity, shape, and shift position provides valuable information for detecting nitrate and understanding its potential interactions with other systems.^40–42^

In an aqueous medium, nitrate exists as a free anion and is likely to form hydrogen bonds with water molecules; these interactions may reduce the anion’s symmetry and partially suppress the Raman-active symmetric vibrational modes.^38^ However, the linear regression between the peak intensity for the scattered symmetric vibrational mode, ν_1_, and the concentration of the anion is the key point in quantitative analysis. Many approaches have been explored to gather analytical information, primarily relying on dynamic experiments and computational analysis of the results; in Figure 4 the integrated peak at 1046 cm^–1^ is reported against the concentration of the nitrate ion in g L^–1^. As an example, in NH_4_NO_3_, the anion–cation interactions have been studied at different temperatures by RS. In the spectra the changes occurring in the N–O deformation modes (symmetric and antisymmetric stretching modes) have been attributed to the weakening of the heteroionic coupling between the NH_4_^+^ and the NO_3_^–^ ions, and have been distinguished in the 2D synchronous and asynchronous Raman spectra (Figure 5). The comparison of the results of both methods enhances the analysis of complex Raman spectra, providing deeper insights into molecular structures, interactions, and dynamics. The 2D synchronous technique identifies coupled vibrations or interacting modes by revealing correlated intensity changes by recording peaks varying simultaneously across two dimensions.^37^

**Figure 4:**
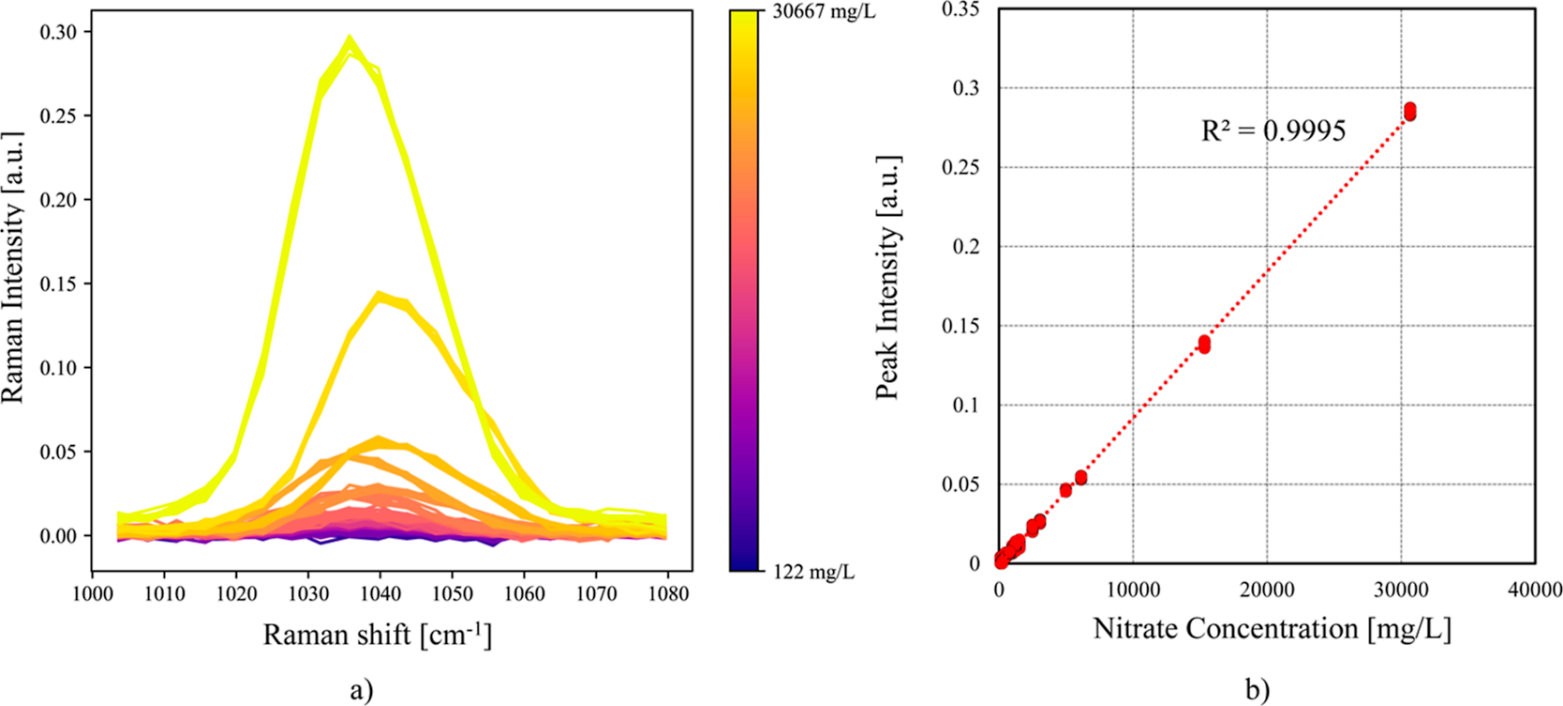
(a) The Raman spectra of Nitrate in its fingerprint region; the baseline of the Raman spectra is first removed, and the spectra are normalized by the max intensity related to the contribution of water; (b) a plot showing the relationship between the peak intensity and Nitrate concentration in mg/L.

**Figure 5:**
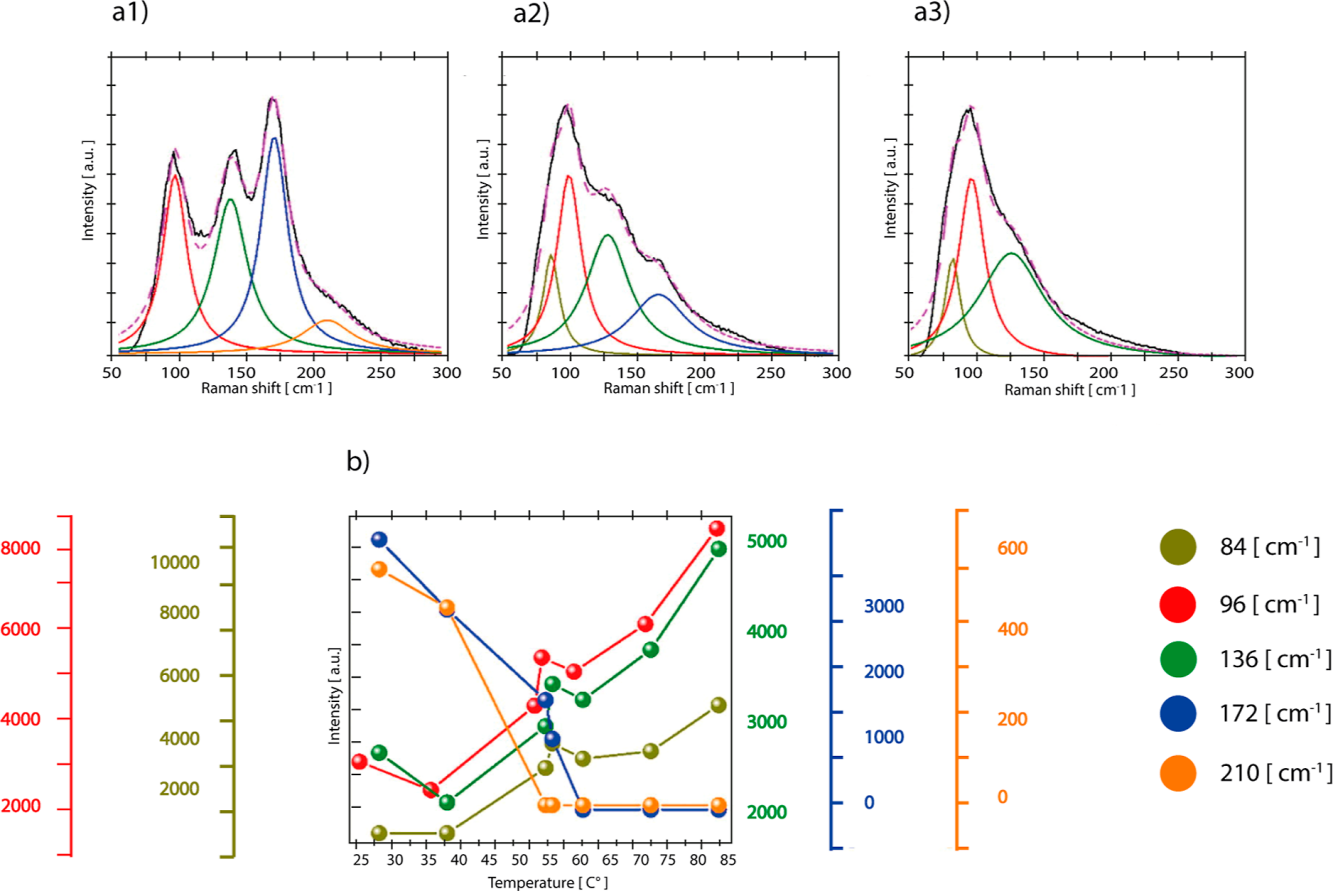
Analysis of low-frequency modes of ammonium nitrate Raman peaks, (a) deconvolution of various low-frequency modes of ammonium nitrate at three different temperatures (a(1) 30 °C, a(2) 54.5 °C, a(3) 80 °C), (b) temperature dependence of the intensity of various low-frequency modes of Raman spectra of ammonium nitrate. Reproduced or adapted with permission from ref 37. Copyright 2021, Elsevier.

Therefore, examining the Raman peak intensity, shape, and shift position provides valuable information for detecting nitrate and understanding its potential interactions with other system (water or cations, for example).^37^ Biswas and Allen^41^ studied hydrated trivalent metal nitrate salts, Fe­(NO_3_)_3_·9H_2_O and Al­(NO_3_)_3_·9H_2_O, in solution and the solid state by Raman and surface selective vibrational Sum Frequency Generation (SFG) spectroscopy. These techniques specifically probe vibrational modes of molecules adsorbed on or near a surface. These methods enhance signals from surface-bound species, allowing detailed investigation of surface chemistry, adsorption processes, and interactions at interfaces. The combination of the surface-specific and chemically sensitive technique with the RS was used to shed light on ion–ion interactions and hydration in several spectral regions spanning low frequency (440–550 cm^–1^) to higher frequency modes of nitrate and water (720 cm^–1^ and 1050 cm^–1^, 1250 cm^–1^ to 1450 cm^–1^, and 2800 cm^–1^ to 3750 cm^–1^). These frequencies span the metal water mode, nitrate in-plane deformation and symmetric and asymmetric modes, and the OH stretch of condensed phase water molecules. As a result, the spectral results indicated a nonlinear water solvation behavior for iron and aluminum nitrates, unlike the linear solvation observed for sodium nitrate; the results are consistent with different solvation behaviors controlled by the concentration.^41^ Furthermore, an additional challenge in Raman detection of nitrate lies in its quantitative analysis, which can be achieved through either direct or indirect approaches.^42^ Continuous research on Raman instrumental setup is currently ongoing to compensate for the low sensitivity of the Raman effect and to achieve easy sampling, fast, and reliable methods for the quantification of nitrate, for example, in drinkable water or natural water basins.^43,44^ In this regard, technicians deal with the fact that drinkable waters should contain nitrate in a law limit that is around 10 mg L^–1^ to 100 mg L^–1^, depending on the national legislation. Hence, Raman instrumental setup should display a Limit of Detection (LOD) upper to these values. The next section reports some RS based methods recently applied for the nitrate analysis.

### Nitrate Detection and Measurement in Water Matrices by Raman Spectroscopy

The early efforts to detect and quantify polluting anions in water, such as nitrate species NO_3_^–^, using RS were documented in the late 1970s.^45^ Furuya et al. established a linear detection range of 6 mg L^–1^ to 100 mg L^–1^ for nitrate ions by dissolving pure NaNO_3_ in deionized water, achieving a LOD of 2 ppm. This high sensitivity was achieved using a 488 nm laser source with a substantial output power of 200 mW, combined with a relatively long acquisition time (1–3 h per spectrum). Additionally, the authors successfully detected and quantified NO_3_^–^ species in other aqueous matrices, such as treated sewage wastewater, within a concentration range of 8 ppm to 32 ppm. These excellent performances were accomplished by mitigating the intrinsic luminescence of the samples via the addition of Potassium Iodide (KI) at a concentration of 5% w/w, which acted as a luminescence quencher. Similarly, Fontana et al. achieved detection and quantification of NO_3_^–^ species in a deionized water solution obtaining a linear range in the 500 mg L^–1^ to 5000 mg L^–1^, by using a Kaiser RXN1 Raman spectrometer with an excitation source at 532 nm (Figure 4). Furthermore, the authors^46^ successfully identified NO_3_^–^ even in the presence of phosphates and sulfates, because the characteristic NO_3_^–^ vibration, centered around 1046 cm^–1^, can be distinguished from the typical vibrations of phosphates at 890 cm^–1^ and sulfates at 982 cm^–1^. Quantitative analysis using RS often faces sensitivity-related limitations; to overcome these challenges, a variety of instrumental and chemical methods are employed.^46^ Jin et al.^47^ showed the application of a selective ion-exchange resin that significantly amplified the standard Raman signal of NO_3_^–^ ions in aqueous solutions. This enhancement facilitated the detection and quantification of nitrate species within the concentration range of 5 mg L^–1^ to 10 mg L^–1^, which aligns with the permissible limits for drinkable water in the United States.^48^ The incorporation of ion-exchange resin significantly improved the LOD, reducing it from 500 mg L^–1^ to 2 orders of magnitude lower, when compared to the direct Raman detection of nitrate species in solution. Kauffmann and colleagues employed RS combined with chemometric methods, including principal component analysis, to identify various ions in aqueous solutions, specifically sulfates, phosphates, chlorides, and nitrates.^49^ Additionally, they utilized Partial Least Squares Regression (PLSR) to effectively estimate the concentrations of NO_3_^–^ within a range of 10 mg L^–1^ to 38.7 g L^–1^ in a multicomponent solution, achieving optimal prediction accuracy at a NO_3_^–^ concentration of 15 g L^–1^. The presence of different ions in solution causes the Raman band’s shift of nitrate, as evidenced in Figure 6 for solutions containing only nitrate, or nitrate and sulfate, or nitrate, sulfate, and nitrite. Additionally, another strategy may involve the use of high-energy laser sources. For example, Ianoul et al. employed deep UV-resonant RS employing lasers with excitation wavelengths at 229 or 204 nm to detect nitrates and nitrites simultaneously in pure water, and real urban sludge samples after biological wastewater treatment.^50^ The method achieved a LOD of 14 μM (approximately 1 mg L^–1^ for nitrates and nitrites) and it demonstrated a linear intensity range corresponding to concentrations from 14 μmol to 3.5 m mol (approximately 1 mg L^–1^ to 220 mg L^–1^ for nitrates and nitrites) in pure water samples. Moreover, the authors conducted direct quantification of both species in real urban sludge samples (post-treatment), successfully quantifying nitrite at a concentration of 0.3 m mol (14.8 mg L^–1^); in the analyzed samples, the NO_3_^–^ species was not present as confirmed by using the Hach method.^49^ Consequently, the use of deep UV-resonant spectroscopy allows the detection of nitrate and nitrite species (and possibly other species), in pure water and real biologically treated samples, because it is possible to enhance the signal intensities of the inorganic species (by excitation at their resonance band) while minimizing fluorescence interferences primarily caused by organic substances dissolved in water. Additionally, the relatively short acquisition time of 10 min renders this system potentially suitable for routine online and offline monitoring of surface water samples.^51^

**Figure 6:**
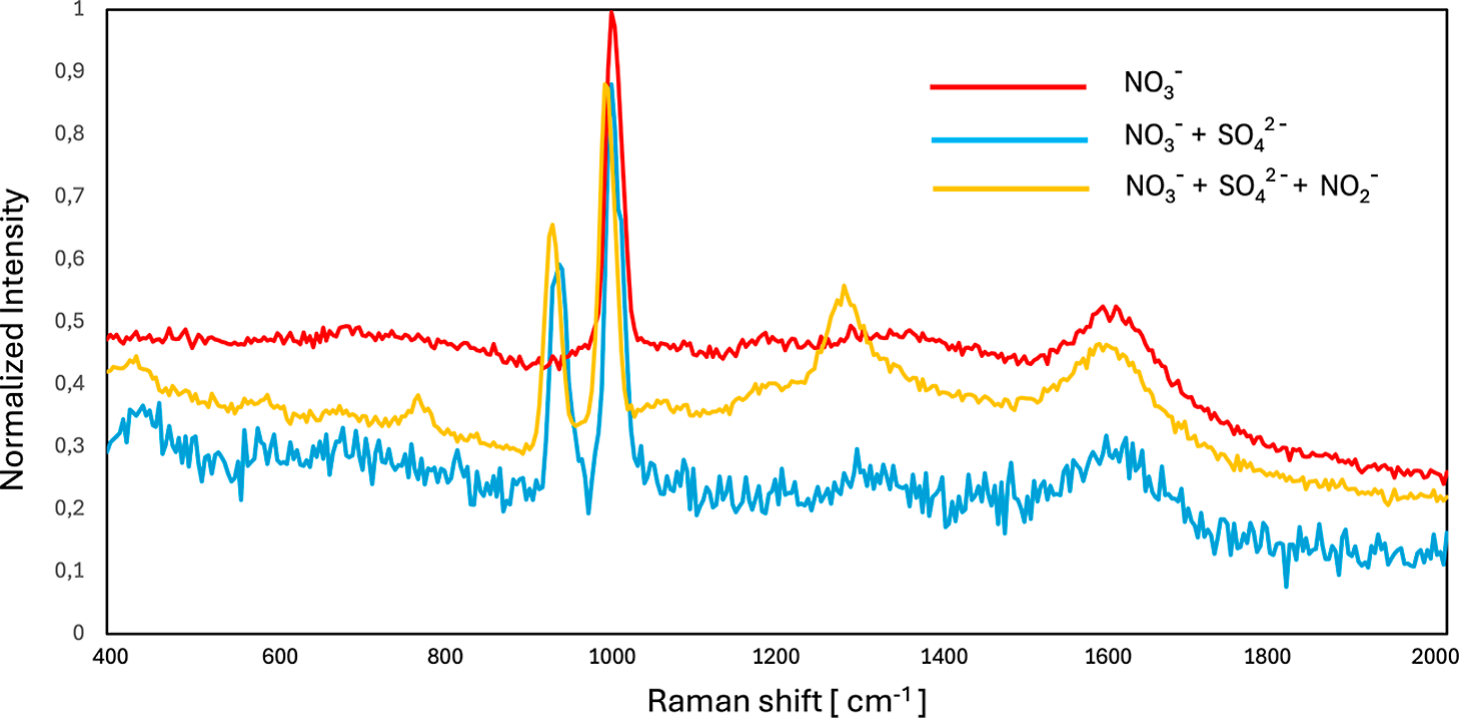
Splitted Raman spectra of aqueous salts solutions with the peaks of the Raman active ions NO_3_^–^, SO_4_^2–^ and NO_2_^–^ around 1000 cm^–1^, recorded for solutions containing only nitrate, or nitrate and sulfate, or nitrate, sulfate, and nitrite. Recorded by Horiba iHR320, 20 mW 532 nm laser, acquisition time 10 s, 5 accumulations.

### SERS Techniques

In addition to instrumental methods, chemical–physical techniques such as SERS have been utilized to improve detection (and quantification) limits in nitrate sensing. In SERS RS, the strong electromagnetic field at the surface of metallic nanoparticles due to plasmonic effects is used to magnify the weak Raman cross-section.^52^ Mosier-Boss and Lieberman utilized SERS with silver nanostructures coated by cationic thiol-based organic ligands to detect and quantify nitrate ions in aqueous solutions, achieving a LOD of approximately 10 mg L^–1^. However, applying this method to real-world samples is challenging due to the presence of similarly sized and charged anions, such as chloride ions. These anions can interact with the charged thiols, displacing NO_3_^–^ ions and thereby reducing the sensitivity of the technique. Furthermore, the sensitivity of this SERS approach is approximately 2 orders of magnitude greater than that of conventional RS, which has an LOD of 260 ppm (260 mg L^–1^) when utilizing a near-infrared laser source (785 nm). Moreover, a near-infrared laser source is advantageous as it limits the luminescence interference in real-world samples.^53,54^ Hu and colleagues^18^ employed a commercially available gold nanostructured system known as Klarite, which consists of gold nanoclusters deposited on a silicon substrate. This system has been shown to serve as an effective SERS substrate for the detection of nitrate ions. The researchers successfully identified NO_3_^–^ with a LOD of 0.5 mg L^–1^, which is approximately 4 orders of magnitude lower than conventional non-SERS Raman techniques. They established a linear detection range from 1 to 10,000 mg L^–1^, facilitating the quantification of NO_3_^–^ in both water and wastewater samples (water containing multiple species), with a percentage error of 8.8%. This error margin is closely aligned with the error percentage found by using standard ion chromatography (5.7% to 7.3%), and the concentration values obtained with both techniques were consistent. Furthermore, the analysis was completed in a total time of just 15 min, utilizing a near-infrared laser at 852 nm, which could be ideal for analyzing real samples due to its nondestructive nature, in contrast to ultraviolet lasers. In addition, a near-infrared laser source also reduces the risk for interference from intrinsic fluorescence, particularly relevant in real-world samples with high content of organic substances.^18^ Almaviva et al.^55^ have recently introduced a SERS method capable of detecting NO_3_^–^ in both deionized and drinking water samples. Their approach contemplated the use of a portable micro-Raman system, which was outfitted with a Gallium Aluminum Arsenide (GaAlAs) diode laser operating at a wavelength of 785 nm with a line width of less than 0.3 nm. The system operated at a power of 150 mW with an acquisition time of merely 10 s for deionized water samples, and at 210 mW with a 30 s acquisition time for drinking water samples. The SERS technique involved incubating water samples with gold nanoparticles at a concentration of 5 × 10^–1^ mg L^–1^. Part of this mixture was then extracted and deposited onto an inert aluminum substrate, which was allowed to dry, thereby concentrating the samples (coffee ring effect). The authors successfully detected [NO_3_^–^] concentrations of 1 mg L^–1^ in deionized water and 19 mg L^–1^ in drinking water (Figure 7). The higher LOD observed in drinking water can be attributed to the presence of competing anions that diminish the availability of SERS “hot spots”, which are critical for the amplification of the NO_3_^–^ signal and the overall sensitivity of the detection method for specific ions.^54^ This technique, nonetheless, enables the detection of NO_3_^–^ ions at concentrations lower than the legal limits.^56^

**Figure 7:**
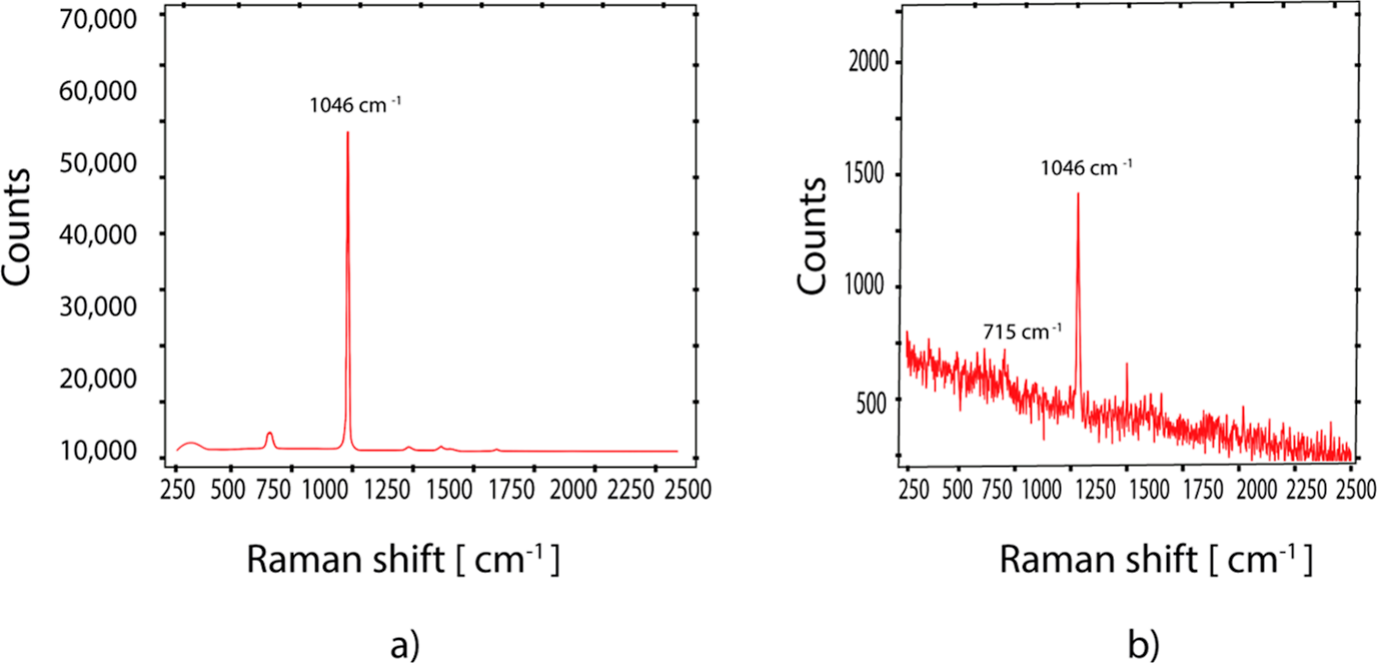
(a) Raman spectrum of ammonium nitrate crystal. Laser spot 40 μm, laser power 150 mW, integration time 10 s (b) Raman spectrum of ammonium nitrate in drinking water (20 mg L^–1^) by applying the protocol of measurements involved scanning the ring-shaped residue formed by the substance in a drop of water (coffee ring) after its evaporation. The volume of the drops was between 2 and 5 μL, with an evaporation time of 15–20 min (RT 20°) and a residual coffee ring of 1–2 mm diameter. In the case of SERS measurements, the drops were deposited in two different ways: (1) on planar, nanostructured SERS substrates or (2) added with an aliquot of suspended gold nanoparticles (Au-NPS), then deposited on the Al-covered microscope glass slide. Laser spot 40 μm, laser power 300 mW, integration time 10 s. Adapted with permission under a Creative Commons CC BY-4.0 from ref 55. Copyright 2022, MDPI.

The methods discussed so far utilize direct detection of nitrate species in solution, by exploring the characteristic Raman-active bands. Additionally, other strategies integrate the increased sensitivity provided by SERS with the selectivity achieved through specific reactions that can identify a particular substance in solution. This approach is especially advantageous for complex mixtures, such as those found in real-world samples, including wastewater, drinking water, and water treated for agricultural purposes. In this context, Correa-Duarte et al.^57^ successfully detected and quantified both nitrites (NO_2_^–^) and nitrates (NO_3_^–^) simultaneously present in real-world samples (spring water, tap water, and plasma) by integrating a modified classical Griess assay with SERS. The authors employed silver Nanoparticles (NPs) functionalized with thiol groups, specifically 4-amino benzenethiol (ABT), and 1-naphthylamine (AC) to react with the NO_2_^–^ species, resulting in the formation of a stable aza-compound, subsequently identified using Resonant Raman Spectroscopy (RSS). Since the (modified) Griess assay detects specifically NO_2_^–^ with the NO_3_^–^ present in aqueous solutions, it was quantified indirectly by running the experiments twice: once analyzing directly the NO_2_^–^ and the second time by reducing all the NO_3_^–^ present in the sample using cadmium pellets. The concentration of NO_3_^–^ was then calculated by subtracting the NO_2_^–^ concentration obtained from the first experiment from the total NO_2_^–^ concentration measured in the second experiment. By illuminating the reaction products at the resonance wavelength of the stable aza-compounds (512 nm), they achieved remarkable sensitivity, with a Limit of Quantification (LOQ) of 2 ng L^–1^, which is nearly 2 orders of magnitude greater than the sensitivity obtained through standard ionic chromatography, which served as a comparative benchmark.^57^ Using RS to detect and quantify nitrates in real samples, especially following the characteristic nitrate signal around 1046 cm^–1^, faces considerable difficulties, particularly at low concentration levels. This difficulty persists even with the employment of nanoparticles and Surface-Enhanced Raman Scattering (SERS) techniques, primarily due to the limited selectivity of gold or silver substrates. In this context, Li et al.^58^ investigate a system in which gold NPs (55 nm diameter) are decorated on their surfaces with thiol-functionalized β-cyclodextrins. These modifications enable the targeted encapsulation of Benzotriazole (BTAH), which is the product resulting from the complete reaction (in an acidic environment) of *o*-phenylenediamine (ODP) with nitrites present in an aqueous solution. SERS signal amplification is achieved when all the BTAH formed is accommodated inside the cavities of thiol-modified β-cyclodextrins. Calibration is performed by considering the intensity of the typical Raman shifts of the BTAH species with different concentrations of nitrite ions. By calibrating in deionized water solutions, the authors found the detection limit at around 0.1 μmol L^–1^ and a linear range was achieved in the range 0.1 μmol L^–1^ to 30 μmol L^–1^ (0.06 mg L^–1^ to 1.8 mg L^–1^) concentrations in laboratory setup (Figures 8 and 9). Finally, after calibration, four kinds of real-world water samples, such as tap water, aquaculture water in fish tanks (carp), aquaculture water in land-based factories (grouper), and seawater, were prepared to validate the present method.

**Figure 8:**
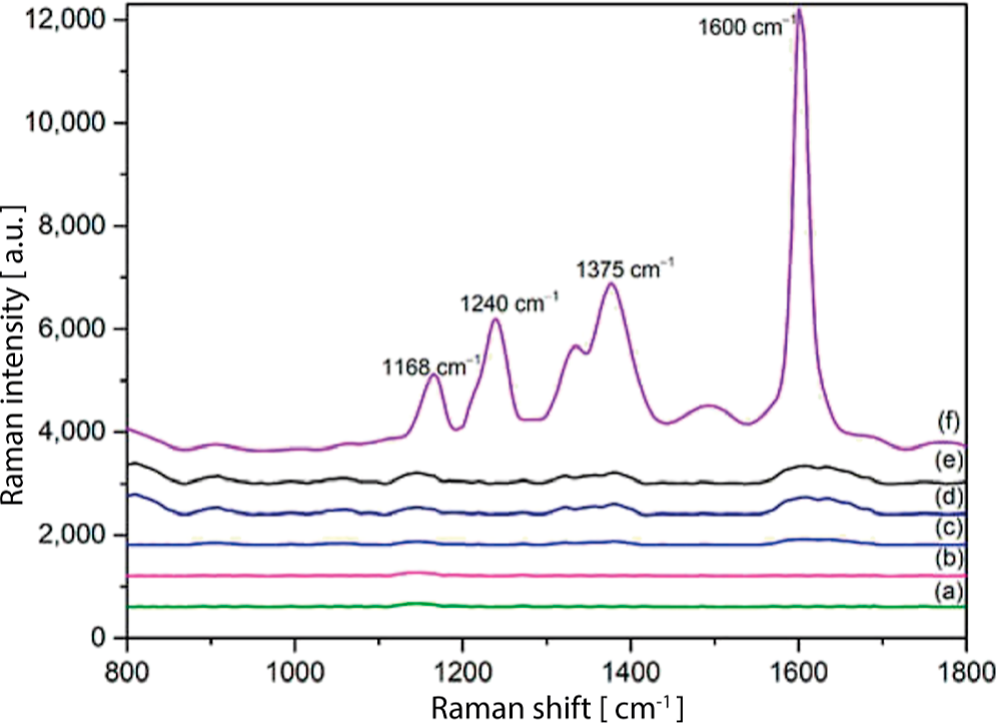
Characterization of the detection process. (a) SERS of OPD (100 μmol L^–1^); (b) SERS of NaNO_3_ solutions (10 μmol L^–1^); (c) SERS of NaNO_2_ solutions (10 μmol L^–1^); (d) NRS of BTAH derived from the reaction between the Nitrite ion and OPD; (e) SERS of BTAH on AuNPs; (f) SERS of BTAH based on SH-β-CD@AuNPs. In this latter spectrum, it is more noticeable Raman characteristic peaks appeared in the SERS of BTAH after mixing with SH-β-CD@AuNPs. Adapted with permission under a Creative Commons CC BY-4.0 from ref 58. Copyright 2024, MDPI.

**Figure 9:**
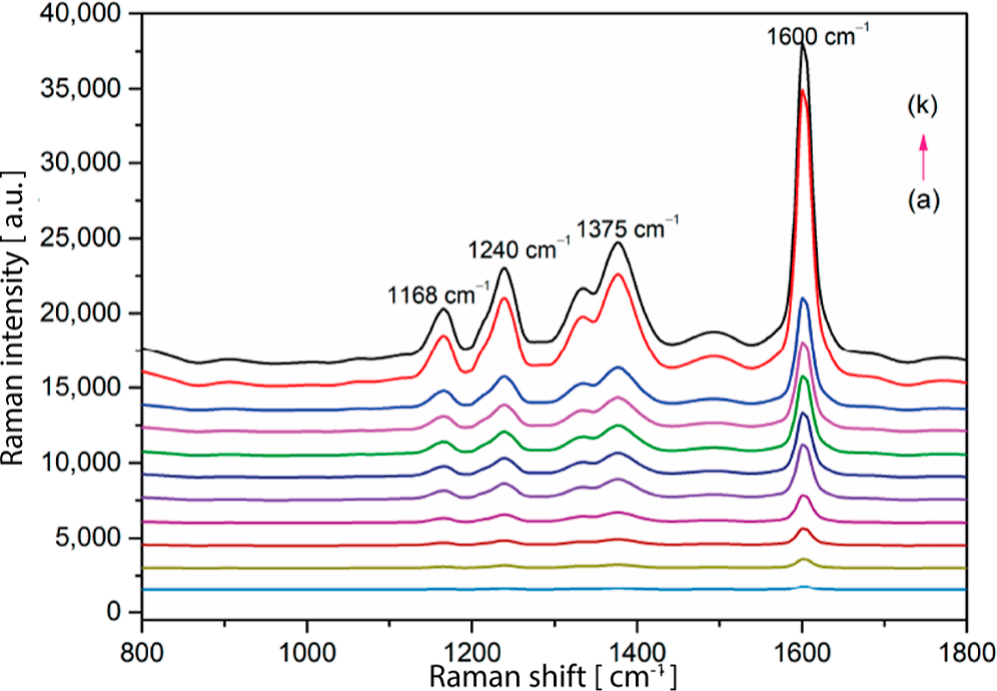
SERS spectra of BTAH at different concentrations of nitrate ions: (a) 0.1, (b) 0.3, (c) 0.5, (d) 0.7, (e) 1, (f) 3, (g) 5, (h) 7, (i) 10, (j) 30, (k) 50 μmol L^–1^. Adapted with permission under a Creative Commons CC BY-4.0 from ref 58. Copyright 2024, MDPI.

The authors found that they could quantify both nitrite (and indirectly the nitrate) concentration in all these real samples, obtaining statistically very close results compared to standard spectrophotometric methods, down to a concentration of 5 μmol (0.3 mg L^–1^) of NO_3_^–^. Since the conversion from the substrate ODP to BTAH species is achieved only when NO_2_^–^ (and not NO_3_^–^) is present in the reaction mixture, to find the NO_3_^–^ concentration, the experiment is performed twice, once analyzing directly the nitrites contained in real samples, and once analyzing the nitrites after the reduction of all nitrates present in solution by reaction with VCl_3_ (reductant). Then, the NO_3_^–^ concentration is determined by subtracting the NO_2_^–^ concentrations obtained in the previous experiments from those in the current set.^58^ In summary, the authors developed a highly sensitive and selective method to quantify both nitrates and nitrites to a concentration down to 5 μmol (0.3 mg L^–1^). However, the method is not particularly fast because it involves the preparation of the gold NPs grafted system and the reaction between ODP and nitrites to obtain the SERS-active species BTAH. Furthermore, to quantify both nitrites and nitrates, it is necessary to perform two sets of experiments, in which in one of the (real) samples all the NO_3_^–^ are converted into NO_2_^–^. The SERS technique is suitable for submicron ambient aerosol particles if aided with electrospray technical support. A new approach to measuring the surface chemical compositions of atmospherically relevant particles was based on surface-sensitive SERS RS by electrospraying Ag nanoparticle aerosols over analyte particles.^59^ Spectral features at υ­(SO_4_^2–^), υ­(C–H), and vibrational modes were observed from the normal Raman and SERS measurements of laboratory-generated supermicron particles of ammonium sulfate and ammonium nitrate mixed with succinic acid or with sucrose. SERS measurements showed strong interaction (or chemisorption) between Ag nanoparticles and surface aqueous sulfate or nitrate. Enhanced spectra of the solid particles revealed the formation of surface-adsorbed water on their surfaces at 60% of relative humidity.

### FERS-Based Raman Spectroscopy Nitrate Detection

FERS combines the unmatched analytical process of RS with the sophisticated use of hollow-core optical fibers. Consequently, FERS is highly selective and sensitive as a label-free, noninvasive, fast optical technique. Hollow core photonic crystal fibers and metal-coated capillaries provide enhancement based on the confinement of laser light.^60^ Unlike the SERS technique, a few works are dedicated to nitrate detection. In a recent paper,^61^ the authors refer to a method based on FERS suitable for solution analysis consisting of liquid-filled capillaries, enabling quantitative measurement of polyatomic anions in solution. In this work, quantitative measurement of nitrate concentrations in water was assessed by multivariate analysis with partial least-squares regression, with a LOD of 8 mg L^–1^ for a measurement time of 30 s, by using FERS in a TeflonAF 2400 capillary. Inside the optical fiber, the laser beam goes in and out of the liquid-filled capillary. The measurement parameters for the acquisition of Raman spectra for calibration and quantitative analysis of nitrate solutions were determined by preliminary characterization and testing of the setup by estimating the signal-to-noise ratio (SNR) of the intensity of the Raman peak of nitrate ions at 1047 cm^–1^. The intensity of the Raman peak was extracted from a fit to the Raman spectrum measured between 921 cm^–1^ and 1950 cm^–1^. The peaks at 1047 cm^–1^ (NO_3_^–^) and that of water at 1637 cm^–1^ (H–O–H bending mode) were simulated and fitted. In Figure 10 the Raman spectrum of a solution of 11.74(5) mM of NaNO_3_ alongside the effect of mathematical elaborations to remove noise and to adjust the baseline are reported. This Raman method was compared with gravimetrically measured concentrations with good agreement and reproducibility. The spectrum was subjected to baseline corrections and deconvolution analysis, leading to a good linear regression between the peak intensity and concentration. Moreover, studies on the effect of laser power and signal-to-noise responses have been correlated. A 10× magnification microscope objective focuses the excitation laser into the capillary and gathers the backscattered Raman light, which is then processed to analyze the Raman peaks.

**Figure 10:**
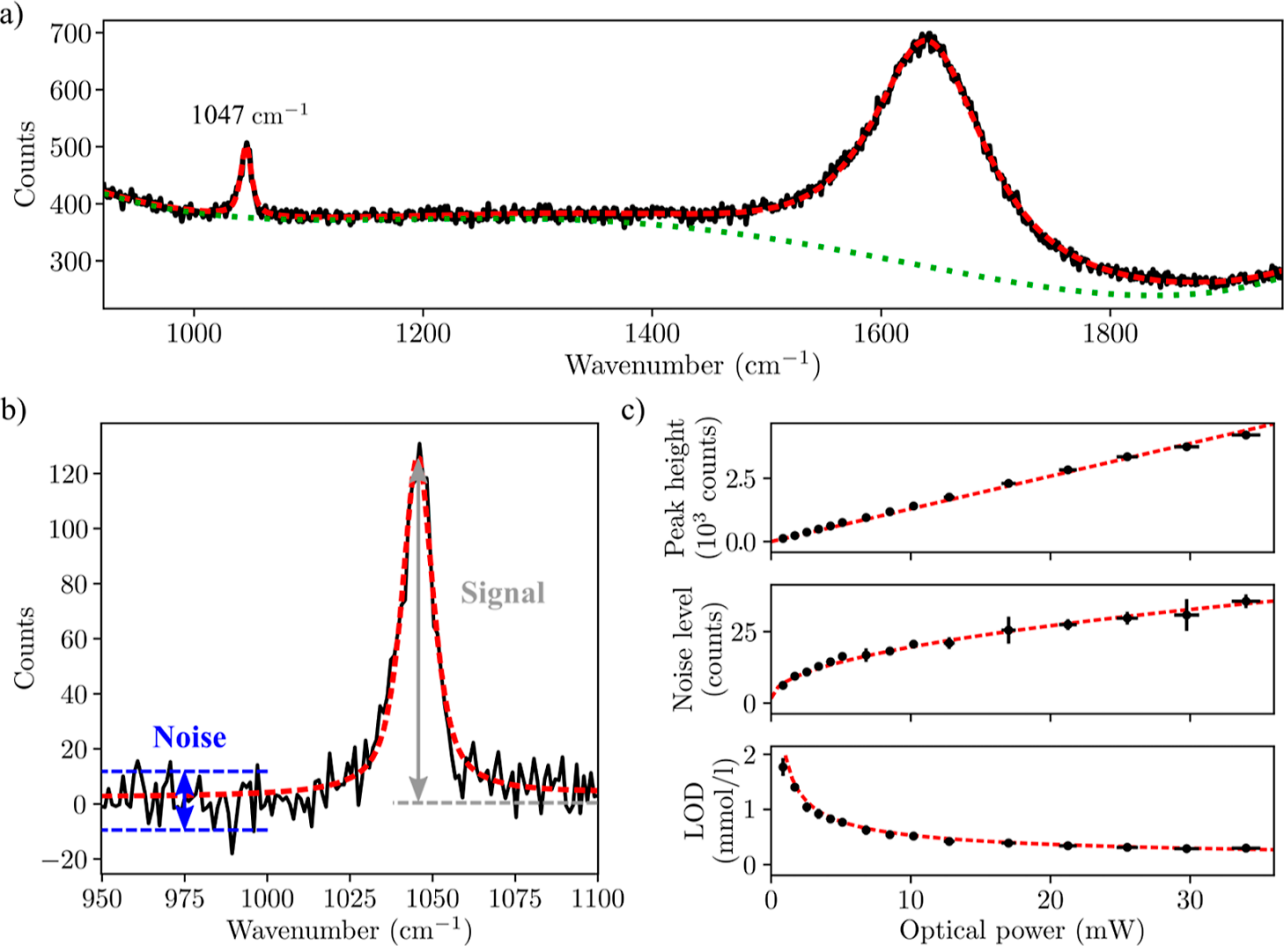
(a) Raman spectrum of a solution of 11.74(5) mM of NaNO_3_ (black line) with fit (red dashed line) and polynomial baseline (green dotted line). Raman peaks of NO_3_^–^ at 1047 cm^–1^ and water at 1637 cm^–1^ (H–O–H bending mode) are fitted with pseudo-Voigt profiles. A zoom-in on the Raman peak of NO_3_^–^ after baseline removal is shown in (b) with a pseudo-Voigt fit (red dashed line). Blue and gray arrowed lines illustrate noise and signal amplitudes, respectively. Optical power at the sample was set at 0.85 m W. (c) Signal, noise, and calculated LOD for measurements of 11.74(5) mM of NaNO_3_ as a function of optical power at the sample. Vertical error bars represent standard deviations estimated from three measurements for each power level. Horizontal error bars correspond to ±3% uncertainty of the power meter reading according to specifications. Adapted with permission under a Creative Commons CC BY-4.0 from ref 62. Copyright 2021, MDPI.

Azkune et al. described a novel FERS platform based on the use of Hydrogel-Core microstructured Polymer Optical Fibers (HyC-mPOF) the Hydrogel-Core microstructured Polymer Optical Fibers (HyC-mPOF).^62^ Sodium alginate was used for hydrogel formation, due to its cross-linking capability with calcium cations to form stable Ca-alginate hydrogel, allowing its complexation once the hollow core of the fiber is filled. The hydrogel is inside the core of the Hollow-Core microstructured Polymer Optical Fiber (HC-mPOF) (see Figure 11) enabling FERS measurements in a functionalized matrix, with high-selectivity Raman measurements. The hydrogel is formed in three complex and delicate steps, and during the Raman spectrum acquisition, the hydrogel formation was continuously monitored and quantified using Principal Component Analysis to verify the coherence between the components and the Raman spectrum of the hydrogel. The study was applied to nickel nitrate detection, which is highly affine for hydrogels or potassium ferricyanide, which is less affine than nickel nitrate. Once the probes had the hydrogel created within their cores, the lower end-faces of the probes were immersed in different solutions for several hours, and Raman spectra were recorded every 5 min. Nickel nitrate exhibits a prominent peak near 1050 cm^–1^; by monitoring this peak during immersion and relating its intensity to the solution’s concentration, the nitrate measurement was achieved.^62^

**Figure 11:**
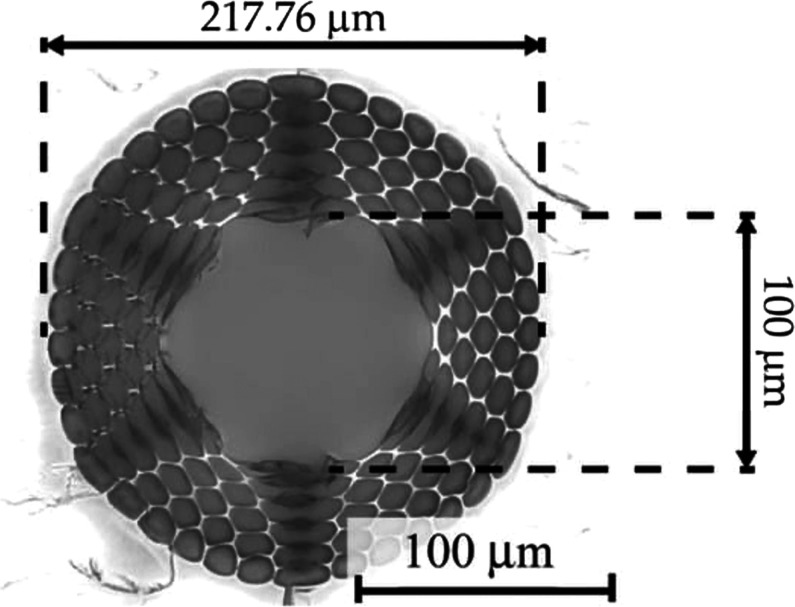
Cross-section microscope picture of the hydrogel-filled optical fiber HC-mPOF microstructure. Adapted with permission under a Creative Commons CC BY-4.0 from ref 61. Copyright 2021, MDPI.

### Machine Learning- and Deep Learning-Based Raman Spectrum Analysis

Recently, ML- and Deep Learning (DL)-assisted RS is a field with growing interest due to the ability of ML and DL algorithms to automatically process the highly complex and dynamic Raman spectrum.^63–65^ The adoption of ML and DL approaches relates to two tasks: classification and regression. In the former, the output is discrete and categorical, whereas for the latter, the output is continuous. An example can be seen in Figure 12.

**Figure 12:**
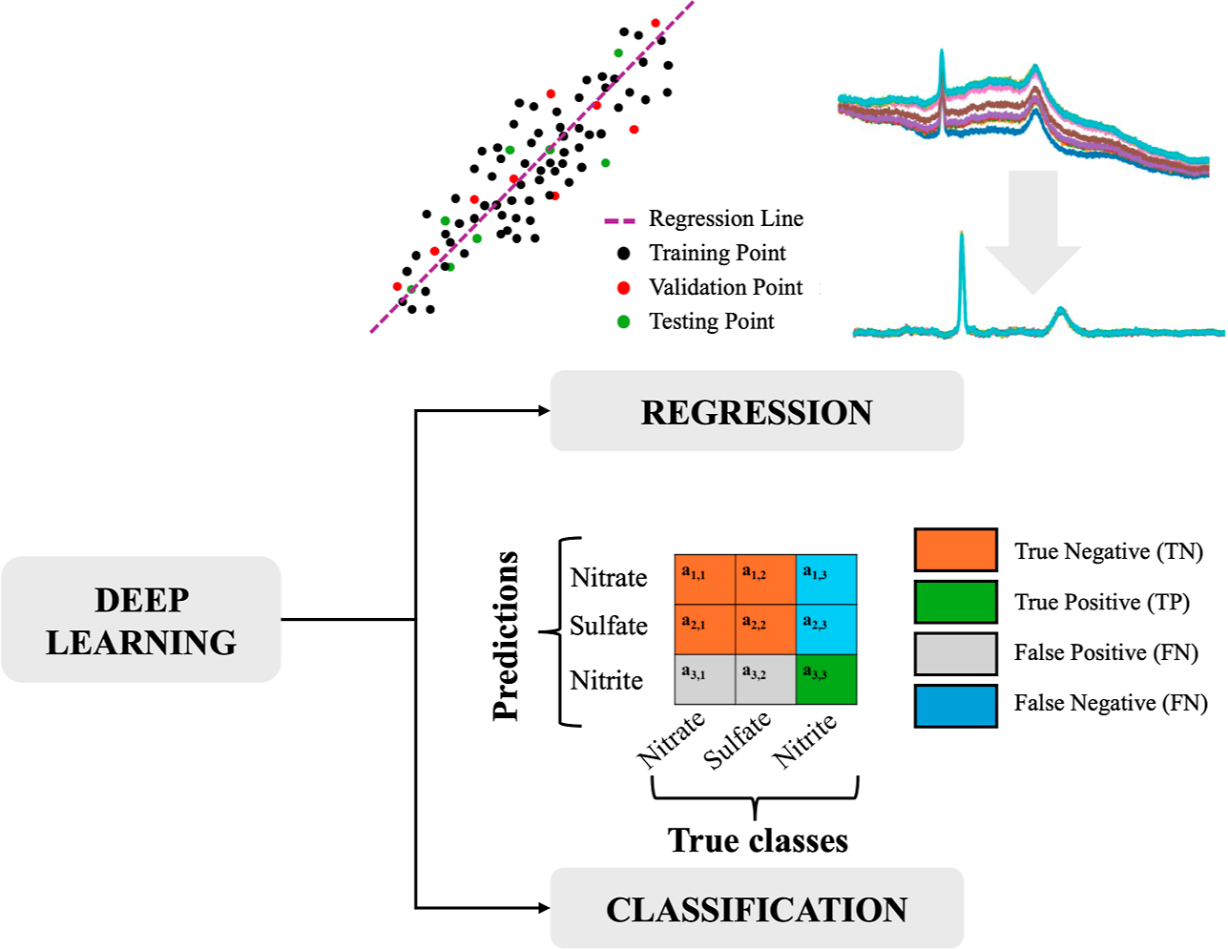
Example of the two main tasks of ML and Deep Learning in Raman spectroscopy.

In the field of Raman analysis, the typical method to recognize the presence of specific analytes in the examined substrate involves a laborious manual pattern matching with an existing database of the wanted analyte. Thus, the requested expertise in RS is high, and there are possibilities of erroneous reading of the spectra. ML is a solution to the problem of objective recognition of substances because it can be trained on large existing data sets of Raman spectra for automatic recognition of substances.^66^

The classification algorithms in artificial intelligence applications to RS are either ML or DL alternatives. ML algorithms are a common choice as they are less complex and require a reduced training time, compared to DL algorithms. On the other hand, they have limitations due to the necessity of an intense preprocessing of the spectra to obtain an adequate result. The preprocessing includes typically cosmic ray removal, smoothing, and baseline correction, followed by feature extraction and dimensionality reduction techniques, such as Principal Component Analysis (PCA). The choice of a baseline correction increases the complexity of the final algorithm. For this reason, DL is often the preferred alternative to remove preprocessing. A DL algorithm can take the raw spectra and learn the proper preprocessing and feature extraction through a training process.

### Overview of ML and DL Techniques for Raman Spectrum Analysis

In this subsection, a brief technical analysis of the typical and most common ML and DL models applied in the RS field is proposed. Starting from traditional ML, an explanation of Linear and Logistic Regression (LR) because they are at the basis of the development of the more complex deep learning models, such as multi-Layer Perceptrons (MLP) and Convolutional Neural Network (CNN), which are thereafter analyzed. For a more comprehensive analysis, the reader can check the source material employed for the explanation.^67^

#### Linear and Logistic Regression

A linear regression is the simplest model available in ML because it aims to find a linear approximation of the target data. Given *n* training examples (*x* ^(0)^, *x* ^(1)^, ..., *x* ^(*i*)^, ..., *x* ^(*n*)^), composed of *m* features $x^{i}={\left[x_{0}^{\left(i\right)},x_{1}^{\left(i\right)},...,x_{j}^{\left(i\right)},...,x_{m-1}^{\left(i\right)}\right]}^{T}$ , a vector of coefficients *w*, then the linear function *h*_*w*_(*x*) is expressed as follows

2 $$y_{pred}=h_{w}\left(x\right)=\sum_{j=0}^{m-1}w_{j}x_{j}$$

Given the observed target values (*y* ^(0)^, *y* ^(1)^, ..., *y* ^(*i*)^, ..., *y* ^(*n*)^), the model aims to find the best coefficients *w* to minimize the residual sum of squares as follows

$$\min_{w}\sum_{i}{\left(h_{w}(x^{\left(i\right)})-y^{\left(i\right)}\right)}^{2}$$ 3

As an extension of the linear regression, it is possible to perform a binary classification by passing the results of the linear approximation through a logistic or sigmoid function σ

4 $$\sigma (h_{w}\left(x\right))=\frac{1}{1+e^{-h_{w}\left(x\right)}}$$

The effect of the sigmoid function is a compression of the possible values between 0 and 1, with two “probability” classes for the binary classification. In this case, the aim is to find the best coefficient to predict the right class; therefore, predict higher values for class 1 and lower values for class 0. The objective function to minimize is usually called binary cross-entropy, and it is as follows

5 $$J\left(w\right)=-\sum_{i}y^{\left(i\right)}\;\log(\sigma (h_{w}\left(x^{\left(i\right)}\right)))+\left(1-y^{\left(i\right)}\right)\;\log(1-\sigma (h_{w}\left(x^{\left(i\right)}\right)))$$

The linear regression can be extended for multiple outputs $y={\left[y_{1},y_{2},...,y_{k},...,y_{K}\right]}^{T}$ by using a matrix *W* of coefficients with dimensions *m* × *K*, with *m* being the number of features and *K* the number of outputs, as follows

6 $$y=h_{W}\left(x\right)={\left[\begin{array}{llll} | & | & ... & |\\ w^{\left(0\right)} & w^{\left(1\right)} & & w^{\left(K\right)}\\ | & | & ... & | \end{array}\right]}^{T}\left[\begin{array}{l} |\\ x\\ | \end{array}\right]=\left[\begin{array}{l} {\left(w^{\left(0\right)}\right)}^{T}x\\ {\left(w^{\left(1\right)}\right)}^{T}x\\ ...\\ {\left(w^{\left(K\right)}\right)}^{T}x \end{array}\right]$$

As a further extension for the *K*-classes classification, the *K*-dimensional output vector *h*_*W*_(*x*) of a linear regression is passed through a softmax layer, which is also the operation performed in larger neural networks for multiclass classification. The softmax operation transforms each result in the *k*th position of the previous operation into Euler’s number *e* to the power of the *y*_*k*_ and divides each element by the sum of all the elements of the vector; it can be defined as follows

7 $$y_{classes}=\frac{1}{\sum_{k}e^{w^{\left(k\right)}x}}\left[\begin{array}{l} e^{{\left(w^{\left(0\right)}\right)}^{T}x}\\ e^{{\left(w^{\left(1\right)}\right)}^{T}x}\\ ...\\ e^{{\left(w^{\left(K\right)}\right)}^{T}x} \end{array}\right]$$

The softmax operation makes each element *k*th of the column the probability that the input data belongs to the class *k*. The generalized cost function to be minimized is a cross-entropy function defined in the following way

8 $$J\left(W\right)=\sum_{i=0}^{N-1}\sum_{k=1}^{K}-\log\left(\frac{e^{{\left(w^{\left(k\right)}\right)}^{T}x}}{\sum_{k=0}^{K}e^{w^{\left(k\right)}x}}\right)\;\text{if}\;y^{\left(i\right)}\;belongs\;to\;the{\;k}^{th}\;class$$

#### Multi-Layer Perceptrons

Multilayer fully connected neural networks, or multilayer perceptrons (MLP), are functions composed of linear and nonlinear operations. The input is a feature vector *x* ^(*i*)^ associated with a label *y* ^(*i*)^, which could be a discrete class or a number. The basic operation of MLP is a single neuron, which is a simple linear regression, followed by a nonlinear activation, which could be potentially any function adding nonlinearity to the operations. The aforementioned sigmoid function is an example of nonlinear activation; however, the most typically employed function to add nonlinearity in neural networks is the Rectified Linear Unit (ReLU), which can be formalized as *f*(*x*) = max­(*x*, 0), meaning that the negative values obtained from a linear regression are set to zero. A schematized representation of a single neuron can be viewed in Figure 13.

**Figure 13:**
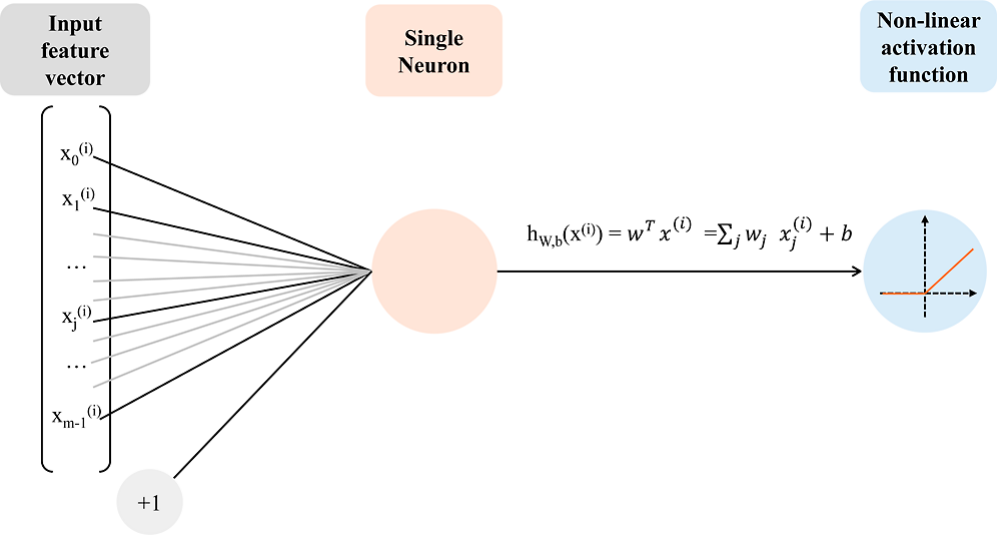
Single neuron; the input feature vector is multiplied by weights and a bias is added; the result is passed to a nonlinear ReLU activation function.

Then, it is possible to increase the number of neurons to *N*, forming a layer of neurons, followed by nonlinear activations. Given an input feature vector *x* ^(*i*)^ with *m* features, it is possible to formalize *N* neurons as a matrix multiplication, in the same way, the simple linear regression is extended to the multinomial case, as follows

9 $$\left[\begin{array}{l} a_{0}^{\left(i\right)}\\ ...\\ a_{N}^{\left(i\right)} \end{array}\right]=a^{\left(i\right)}=ReLU\left(W^{T}x^{\left(i\right)}\right)=ReLU\left({\left[\begin{array}{llll} | & | & ... & |\\ w^{\left(0\right)} & w^{\left(1\right)} & & w^{\left(N\right)}\\ | & | & ... & | \end{array}\right]}^{T}\left[\begin{array}{l} x_{0}^{\left(i\right)}\\ ...\\ x_{m-1}^{\left(i\right)} \end{array}\right]\right)$$

The output of the *N* neurons can then be given as input to another layer of neurons performing the same linear matrix multiplication followed by a nonlinear activation, and this can be repeated to a higher order of complexity. A schematized example can be seen in Figure 14.

**Figure 14:**
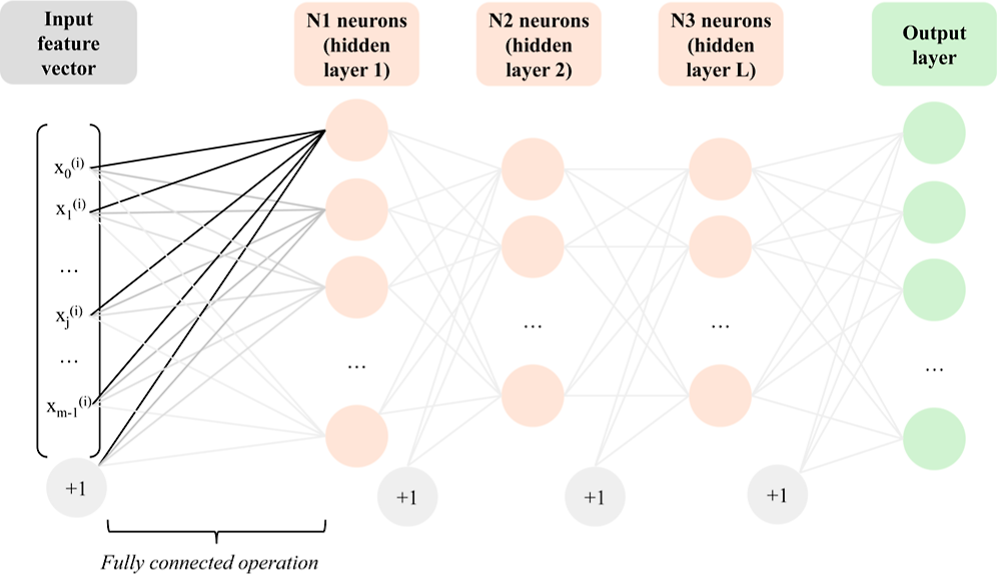
Deep Multilayer perceptron. The input feature vector passes through L hidden layers with *N*_*l*_ neurons each. As a visual simplification, the nonlinear activations are not visible, but they are applied after each layer. Eventually, an output layer with the desired dimension gives the results of the neural network. If a classification is performed, the last values go through a softmax activation layer.

The process by which the neural networks learn the adequate weights and biases to perform their basic operation is an optimization problem where an objective function is minimized. The typical algorithm employed for the optimization is the stochastic gradient descent coupled with a backpropagation of error algorithm.

#### Convolutional Neural Networks

The issue with the previous architecture is its fully connected nature, which leads to a rapid growth of the number of operations performed with high-dimensional inputs as images. Consequently, to deal with images, CNNs appeared as a solution, where the basic operation is a convolution filter, which is a locally fully connected operation. The convolutional filter is a set of weights and bias acting on a local patch of an image or signal, performing a fully connected operation on that patch. Then, the filter shifts to adjacent patches to produce the final feature map. Given a large matrix *I* of dimensions *R* × *C*

10 $$I=\left[\begin{array}{lllll} a_{1,1} & a_{1,2} & a_{1,c} & ... & a_{1,C}\\ a_{2,1} & a_{2,2} & a_{2,c} & ... & a_{2,C}\\ a_{r,1} & a_{r,2} & a_{r,c} & ... & a_{r,C}\\ a_{R,1} & a_{R,2} & a_{R,c} & ... & a_{R,C} \end{array}\right]$$

a convolutional filter *K*, defined by weights and bias (*w*, *b*) operates on a smaller matrix or patch of dimensions *R*′ × *C*′, as follows

11 $$K=\left[\begin{array}{lllll} w_{1,1} & w_{1,2} & w_{1,c^{\prime }} & ... & w_{1,C^{\prime }}\\ w_{2,1} & w_{2,2} & w_{2,c^{\prime }} & ... & w_{2,C^{\prime }}\\ w_{r^{\prime },1} & w_{r,2} & w_{r^{\prime },c^{\prime }} & ... & w_{r^{\prime },C^{\prime }}\\ w_{R^{\prime },1} & w_{R^{\prime },2} & w_{R^{\prime },c^{\prime }} & ... & w_{R^{\prime },C^{\prime }} \end{array}\right]$$

12 $$a_{r^{\prime },c^{\prime }}^{\prime }=\sum_{r=0}^{R^{\prime }}\sum_{c=0}^{C^{\prime }}a_{r,c}w_{r,c}+b$$

CNNs are composed of a series of convolutional kernel operations that act as filters of the input raw data. The kernel learns how to filter, preprocess, and extract the necessary features to achieve the final task. A schematized representation of the convolutional filter operation is in Figure 15.

**Figure 15:**
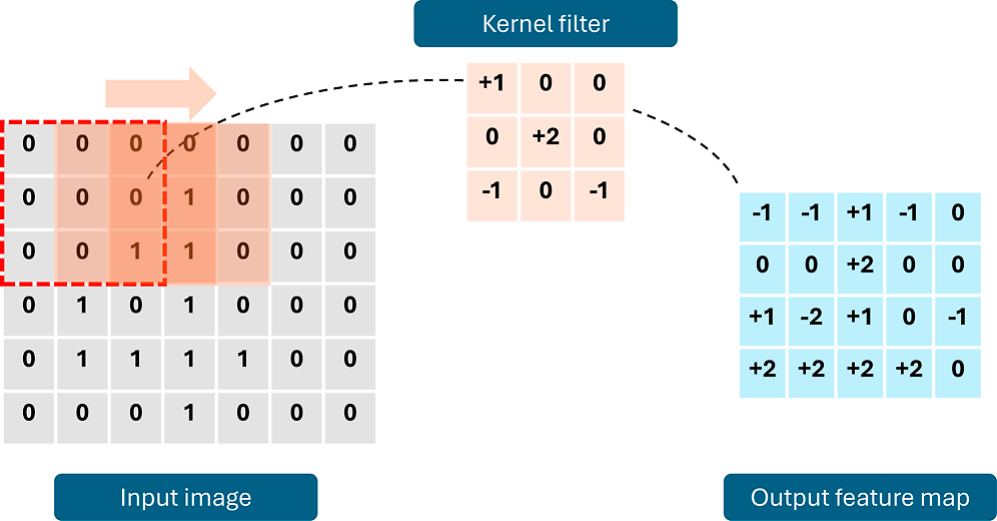
Convolutional kernel operation.

Usually, the kernel blocks are followed by max-pooling operations, nonlinear activations, such as the aforementioned ReLU, and batch-normalization.^68^ The max-pooling operations downsample the output by choosing, in a sliding window, that is smaller than the overall output feature map, the highest value in that window.

Even though CNNs were initially proposed to handle images, their ability to capture local patterns can be translated to one-dimensional input, such as in the case of Raman spectra, by using a one-dimensional kernel. A schematized example of 1D-CNN can be seen in Figure 16.

**Figure 16:**
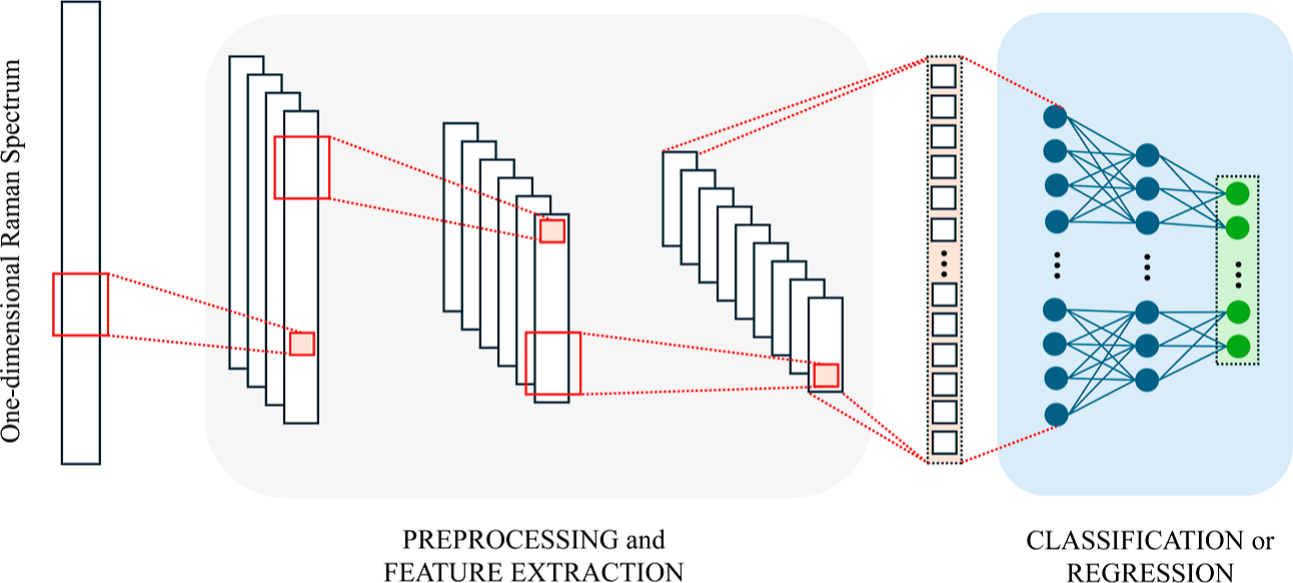
Example of 1D CNN. The red rectangle represents the convolutional filters, usually followed by a max pooling operation to reduce the dimensionality of the data and a nonlinear activation, such as a ReLU.

### Recent Trends in Deep Learning-Based Raman Spectroscopy

The recent trend is to employ one-dimensional CNN as shown in Figure 16 to process and classify Raman spectra. One of the first efforts to introduce 1D-CNN in the field of applied RS is given by the approach proposed by Liu et al.,^66^ where the DL approach is compared to typical ML algorithms with improved performance for both baseline-corrected and raw spectra on the RRUFF data set^69^ with top-5 accuracy as high as 96.3% over 1671 different classes and top-1 accuracy of 88.4%.

Nevertheless, the deep learning approach is data-driven and needs many examples to produce adequate results. Consequently, it is not an easily accessible method for novel fields that should start a massive data collection process. For example, the RRUFF data set is one of the largest existing Raman data set with 5168 spectra and 1671 classes of minerals, therefore it is often used to train and test 1D-CNNs with success,^66,68^ but it is the result of a multiyear project to which different university worldwide are collaborating.

The issue of having a small data set is usually solved by employing transfer learning, data augmentation, or both. The topic of transfer learning was deeply investigated in two recent reviews regarding ML and deep learning approaches in applied RS.^63,64^ Transfer learning in the field of deep learning can be broadly defined as improving the capacity of a learner neural network by transferring information from a related domain.^70^ Using the taxonomy of a recent review on deep transfer learning,^71^ it is possible to distinguish three modalities for network-based transfer learning as can be observed in Figure 17.

**Figure 17:**
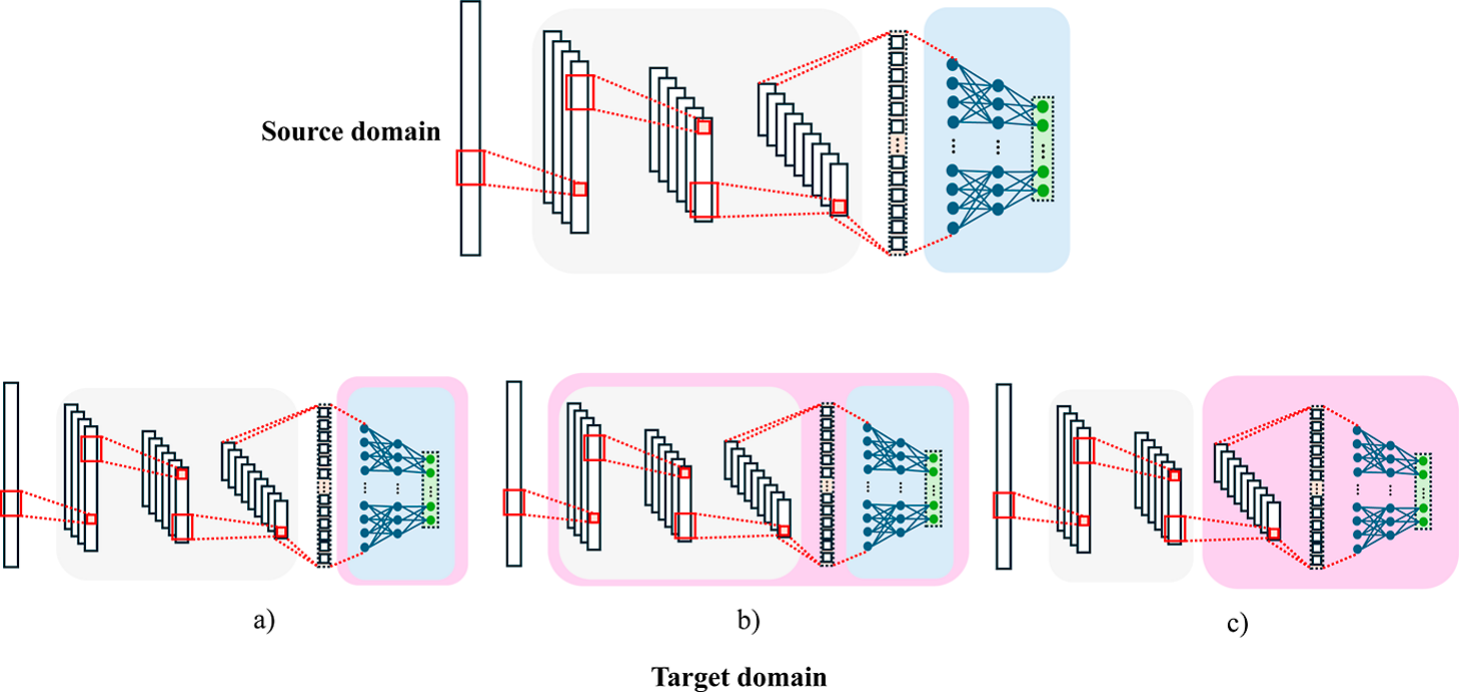
Transfer learning modalities.^71^ (a) Freezing describes the transfer of pretrained model weights to a new model where only the last classification/regression layers are trained from scratch on the target domain. (b) Fine-tuning describes the process of fine-tuning where all the pretrained model weights are updated by training on the target domain to achieve better performance. (c) Progressive learning describes the method of freezing a part of the network to maintain important feature extractors and then adding new layers trained from scratch on the target domain.

The great advantage of transfer learning is that it is possible to transfer knowledge acquired by a network over a large source domain data set and pass it down to a novel task and target domain, solving the issue of small examples in the target domain.

Synthetic data set generation or data augmentation is another way to address the challenge of data scarcity affecting this field. Synthetic spectrum generation aims to build a data set from scratch, replicating the common appearance of Raman spectra by producing a predetermined pattern of Gaussian profiles with different heights, widths, and mean wavelength corresponding to a class of analyte.^72^ Usually, this generation is employed to validate and benchmark different model performances or to pretrain a model to recognize patterns in a spectrum. Once a spectrum is generated, peak heights, widths, and positions are slightly altered by introducing noise to replicate the distortions caused by instrumental artifacts. Some alterations could include adding a baseline, dark noise, and cosmic ray spikes.^73^ On the other hand, data augmentation is a technique employed to increase the training data set size starting from known spectra that are slightly altered to produce meaningful variations. Common ways to augment training set size are the addition of noise, slight wavelength and peak shifts, and summation of different spectra.^74^

### ML-Based Raman Spectrum Analysis on Nitrates in Water Samples

Regarding identification of Nitrates, Raman spectrum analysis is applied to two main studies,^74,75^ where a 1d-CNN replicated from^66^ and trained on the RRUFF data set was fine-tuned on a small set of acquired spectra to improve the performance of detection of many substances, including Nitrate. Even though the typical preprocessing of Raman spectra can be avoided when using a DL approach,^66^ both studies include a standard processing pipeline with baseline correction, spectra smoothing using the Whittaker-Henderson algorithm, cosmic ray spike removal, normalization from 0 to 1, and removal of noisy spectra. Regarding the regression of the concentration of nitrate, there is only one preprint study,^76^ showing the recency of the research line on Nitrates’ concentration regression; the study employs SERS and 2D-CNN to a bidimensional representation of spectra obtained by relative position matrix combined with the normal one-dimensional spectra processed by a Long–Short-term Memory.

Given the recency and the limited amount of works on Nitrate detection and quantification through Raman measurements and ML, there is still a gap in the scientific literature concerning the possible steps to perform to achieve either a classification or a regression. Thus, we propose possible pipelines based on previous works in complementary and similar fields that could be applied to Nitrate analysis. Before using any learning algorithm, a data collection phase is necessary, recording the examples needed to train, validate, and test the algorithm. The data set, composed of the different spectra, should first be divided into a training set, a validation set, and a test set. The training set is composed of a collection of examples and their labels (e.g., class or concentration of a substance) that are given to the learning algorithm to produce an adequate predictor with the optimal weights and biases to minimize an objective function. Then, the validation set is employed to tune the choices of hyperparameters, which can be chosen by the user. They include the type of architecture, the number of layers, the dimensions of the layers, and parameters such as the learning rate and the number of iterations or epochs of update during the optimization of weights. Eventually, a testing set composed of spectra that are not seen by the learning algorithm and the user is employed to produce generalized metrics on new, unseen data.

For what concerns the algorithms, a summarization of the possible pipelines from the input raw Raman spectra to the wanted output is presented in Figure 18.

**Figure 18:**
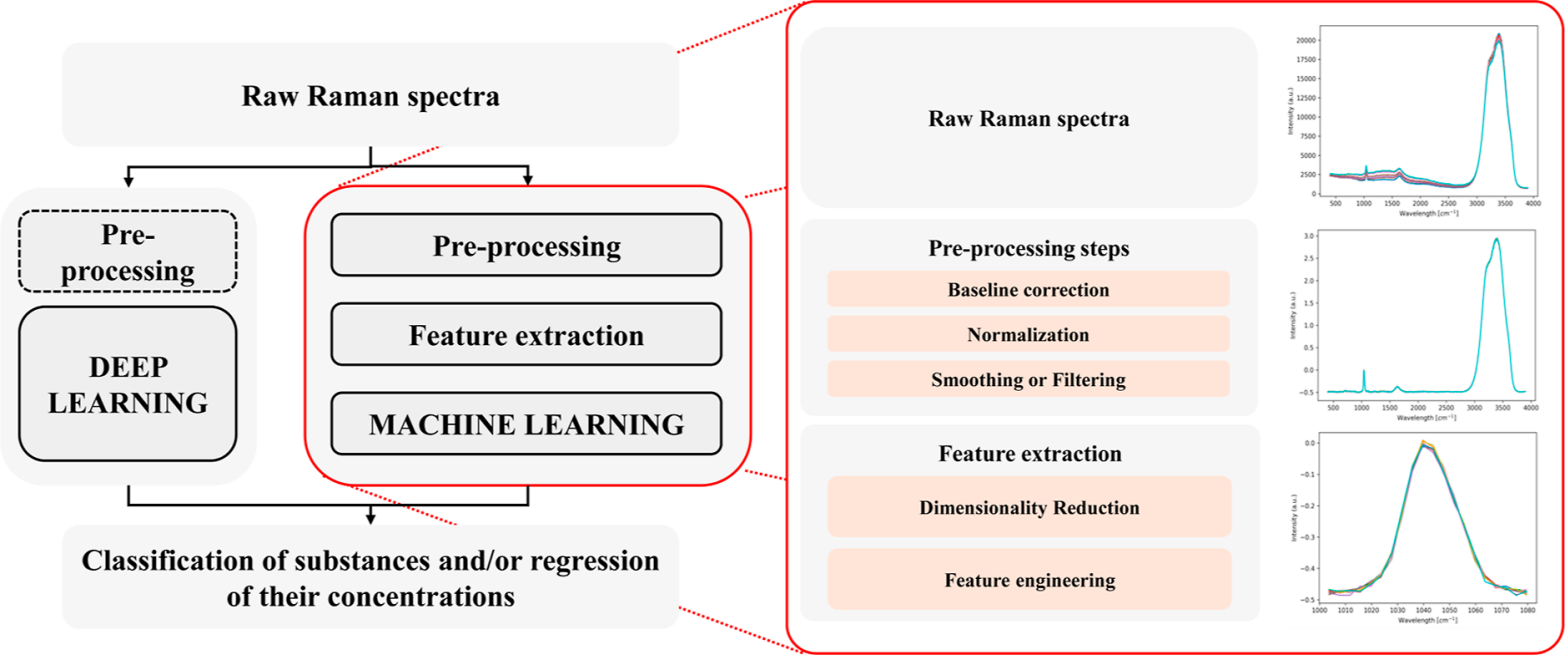
Steps from raw Raman spectra to classification and/or regression of substances. Deep learning methods could include preprocessing for limited data availability to improve model stability or to transform the input one-dimensional spectra to a two-dimensional representation handled by validated computer vision algorithms. On the other hand, machine learning needs preprocessing steps and a feature extraction step, which reduces the dimensionality of the input data.

For deep learning, the preprocessing steps are optional and dependent on the size of the training data set. Deep learning approaches can potentially be trained to implement all the steps from the preprocessing to the feature extraction and final classification or regression. For machine learning, the complexity of the spectra should first be reduced with a preprocessing method, including baseline correction, normalization, and smoothing; then, the high dimensionality of the spectra should be reduced with a feature extraction step, which could include a crop to highlight certain regions of the spectra (e.g., the fingerprint region of the Nitrate), a Principal Component Analysis and a feature engineering step; eventually, a machine learning model can be chosen to maximize the performance of classification and/or regression.

The choice between ML and deep learning techniques depends on the data availability, the expertise of the users, and the needed interpretability. The performance improvements depending on the amount of data for machine learning against deep learning alternatives can be viewed in Figure 19.As an initial approach, ML is suggested in conditions of data scarcity and if the users can process the spectra in such a way as to remove distortions and extract relevant features. The choice of the preprocessing steps and features can be made either by knowledge of the field or by leveraging an optimization function and a search method in a given set of possible preprocessing algorithms. Additionally, the recent diffusion of Python libraries, collecting different state-of-the-art processing techniques, such as RamanSPy,^78^ and integrating the possibilities of ML development, is accelerating the research toward the use of novel intelligent algorithms for Raman analysis. The preprocessing steps aim to reduce the variability in repeated measurements by Raman spectroscopy to help the ML models produce more stable results. An example of this process is reported in ref 79, where a grid search and a genetic algorithm are used to choose the best combinations of preprocessing steps that could minimize a metric of spectra variability or maximize the final accuracy of the ML model. Furthermore, with a ML approach, the user has more control over all the processes from the raw input to the outcome, and depending on the choice of the model and the dimensionality of the feature space, the possibility to interpret the results.

**Figure 19:**
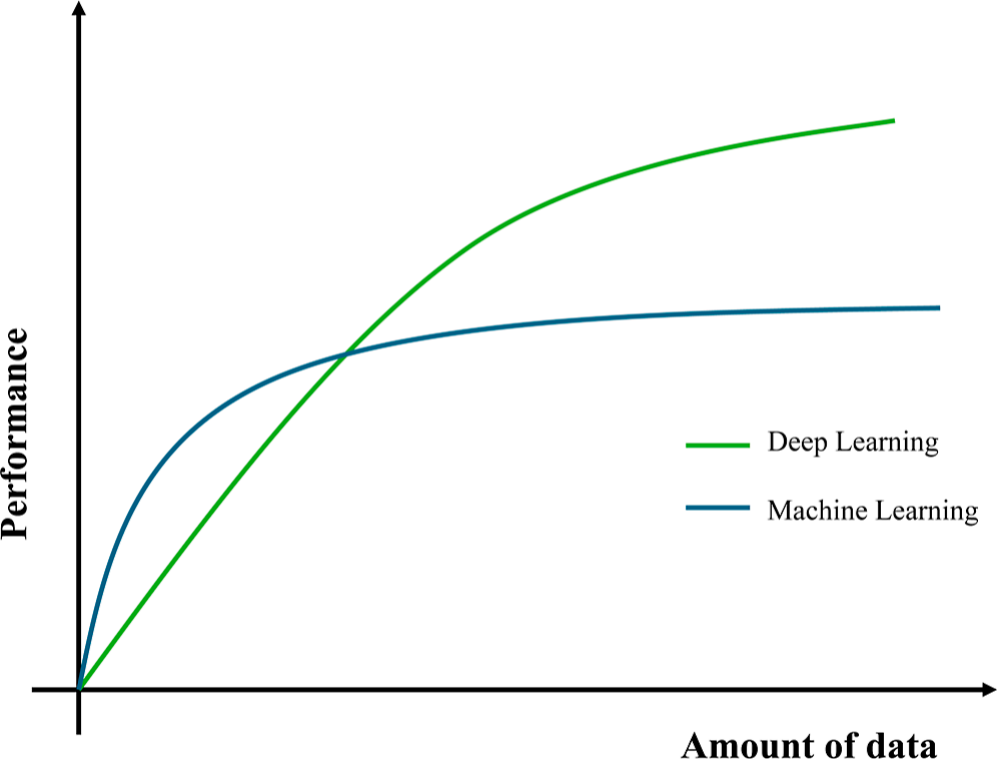
Performance of ML and DL with increasing amount of data in the training set. Adapted with permission under a Creative Commons CC BY-4.0 from ref 77. Copyright 2019, MDPI.

DL could be an option if the data set is larger, having the advantage of a reduction or absence of preprocessing steps. On the other hand, more complex architectures need greater computational resources for both the training phase and the deployment phase, leading to possible concerns when aiming for real-time processing of the spectra. Moreover, the interpretability of DL techniques is usually limited to the visualization of heatmaps of activation layers.

## Discussion and Future Directions

This review considers the case study of nitrate detection and concentration measurement in water by RS. A general description of RS features this technique with the following remarkable key bullets: it is a light scattering technique with scatters related to molecular vibrational modes, is generally poorly sensitive, gives highly resolved spectra, is sensitive to fine structural molecular changes, is suitable for aqueous samples, needs easy sampling, affords nondestructive analysis, displays wide versatility, exhibits many applications by infinite instrumental components matches, applies from micro to macro analysis, measures through transparent containers allowing flow analysis. Remarkably, the intensity of the scattered light strongly depends on both the power and wavelength of the laser, the light path thickness, and the molecular cross-section, hinting linear correlation between the intensity and the concentration. The symmetric vibrational mode of nitrate anion is Raman active and is detected by this technique. Besides, in situ water analysis, flow water analysis, or prompt analysis by handling instruments in sudden situations are current targets in the research of innovative methods for detecting and measuring nitrate (or other pollutants) in aqueous matrices. In this review, some RS instrumental setups aimed to enhance the sensitivity of the Raman technique in nitrate detection are described. The Table 1 reports a list of the main papers herein discussed, the results in terms of LOD and LOQ, and brief notes of the technical setups.

**Table 1:** Main Investigation on the Application of RS to the Detection and Measurement of Nitrate in Water

ref.	year	analyte	LOD/LOQ	technical details	SERS	FERS
^45^	1979	NaNO_3_	2 ppm (2 mg L^–1^)	488 nm, 200 m W, 1–3 h for acquisition time, macro Raman	×	×
^45^	1979	NO_3_^–^	8–32 ppm depending on the sample (8–32 mg L^–1^)	488 nm, 200 m W, 1–3 h for acquisition, addition of KI as luminescence quencher	×	×
^46^	2012	NO_3_^–^ with PO_43_^–^ and SO_42_^–^	500 mg L^–1^ (linear range 500–5000 mg L^–1^)	532 nm, Kaiser RXN1 Raman, micro Raman	×	×
^47^	2022	NO_3_^–^	5–10 mg L^–1^	enrichment of the sample by selective ion-exchange resin	×	×
^49^	2013	NO_3_^–^, PO_43_^–^, SO_42_^–^, and Cl^–^	LOD 10 mg L^–1^ (linear range 10 mg L^–1^–38.7 g L^–1^)	RXN-1 Raman spectrometer 532 nm excitation source	×	×
^51^	2024	NO_3_^–^	1mgL^–1^ (linear range 1 mg L^–1^–220 mg L^–1^ linear range)	229 and 204 nm deep UV laser (resonant Raman), 10 min acquisition time, micro Raman	×	×
^52^	2012	NO_3_^–^ and SO_42_^–^	LOD 10 mg L^–1^ with SERS and 260 mg L^–1^ with normal Raman	Macro Raman 852 nm laser power source, 50 m W output power	√	×
^18^	2013	NO_3_^–^ and SO_42_^–^	LOD 0.5 mg L^–1^ (linear range 1 mg L^–1^–10 000 mg L^–1^)	Renishaw RM1000 micro Raman spectrophotometer (Gloucestershire, UK) equipped with a Leica DMLB microscope (Wetzlar, Germany) and a 785 nm near-infrared diode laser source, at 10 s exposure time and 30 m W laser power	√	×
^55^	2022		LOD 1 mg L^–1^ in deionized water and 19 mg L^–1^ in drinking water	micro-Raman system “i-Raman” by Bw&Tek. 150 m W, 10 s acquisition time for deionized water samples, and at 210 m W with a 30 s acquisition time for drinking water samples, 785 nm laser source	√	×
^57^	2015	NO_3_^–^ and NO_2_^–^	LOQ 2 ng L^–1^	micro Raman instrument: micro–Renishaw InVia Reflex system with excitation wavelength 514 nm	√	×
^58^	2024	NO_3_^–^ and NO_2_^–^	LOD 0.06 mg L^–1^ in laboratory setup and 0.3 mg L^–1^ in real-world samples	micro Raman. QE-Pro Raman spectrometer, 785 nm laser source, 300 m W laser source, 5 s acquisition time	√	×
^61^	2021	NO_3_^–^	LOD 0.13 mM (8 mg L^–1^)	micro Raman Horiba iHR320 20 acquisitions of 1.5 s power of 40 m W at the sample and a capillary length of 89 m m, 532 nm laser excitation source	×	√
^62^	2021	NO_3_^–^	nd	Renishaw (Gloucestershire, UK) inVia confocal Raman Microscope. The excitation wavelength of the laser was 785 nm, and the power launched to the sample was 27.61 m W for 10 s for each measurement. With this power and time exposition, any thermal effect on the sample or fiber probe was discarded. The spectra were recorded from 100 cm^–1^ to 3200 cm^–1^ using a 20× objective with a 0.4 NA from Leica (Wetzlar, Germany).	×	√

In general, the proposed solutions display good sensitivity, ranging from a few to hundreds of mg L^–1^. The main strategies to increase the nitrate signal intensity are based on (i) pretreatment of the sample to enrich the nitrate concentration by, for example, ion-exchange chromatography, (ii) selection of low wavelength lasers, (iii) usage of high-power lasers (iv) increase of the acquisition time until hours, (v) application of surface materials acting as signal enhancers SERS and/or (vi) of special instrumental assemblies using, for example, specialized optical FERS, (vii) chemical quenching of fluorescence. So far, the best results in terms of detection sensitivity and peak intensity/concentration linear regression are represented by those cases matching high-energy UV laser and Resonant Raman spectroscopy (UVRRS) or Surface Enhanced Raman Spectroscopy. For these latter, linear relationships between peak intensities and concentration were found both in laboratory tests and even in wastewater sludges. Unfortunately, using UV excitation, an increase in Raman signal intensity can be achieved with shorter acquisition times, but molecules excited by UV laser light frequently emit fluorescence that far surpasses the Raman signal. However, the hardware of a UVRS instrument requires expensive special optics and fibers suitable for this high-energy light, downgrading this choice. In the last part of the review, the aid of computational AI methods in data processing has been considered too. ML methods have been taken into consideration. Beyond the classic data processing (baseline correction, noise removal, smoothing), data classifications or regression operations by ML might be useful to refine sets of data or to obtain indirect parameters such as concentration, respectively. This field of research has just opened; few works have been dedicated to the applications of these methods in Raman spectroscopy, and just one has been dedicated to the classification analysis of minerals with nitrate anion. A significant challenge in applying these methods appears to be the availability of large data sets and the selection of appropriate algorithms. However, considering the substantial potential of ML techniques, optimizing the signal-to-noise ratio in remote sensing responses for nitrate analysis seems to be a promising strategy that could be extended to other analytes as well.

## Conclusion

The various technical approaches outlined above demonstrate that a broad range of analytical methods exists for detecting nitrate ions, leveraging the combination of instrumental techniques with chemometric methods and/or chemical treatments to eliminate any interfering effects. Indeed, even when focusing on a single analyte, such as the nitrate ion, remote sensing does not lead to a narrow set of solutions but rather reveals a wide range of possibilities, each with its own value and scope for analysis. In this context, ML techniques—both for classification and as “intelligent” tools for processing output data—can add significant value to Raman spectroscopy-based analysis. First, it has been shown that ML can more effectively extract meaningful information from spectral data, opening new avenues for analytical science and consolidating dispersed data related to a specific analyte into a valuable knowledge base accessible to many. Second, by leveraging existing data, ML can enhance the processing of Raman signals, thereby improving detection capabilities. To achieve this, large Raman data sets are necessary to train the ML models effectively.
